# Resting State Cortico-Limbic Functional Connectivity and Dispositional Use of Emotion Regulation Strategies: A Replication and Extension Study

**DOI:** 10.3389/fnbeh.2020.00128

**Published:** 2020-07-28

**Authors:** Denise Dörfel, Anne Gärtner, Christoph Scheffel

**Affiliations:** Faculty of Psychology, Technische Universität Dresden, Dresden, Germany

**Keywords:** emotion regulation, resting-state, amygdala, preregistration, cortico-limbic connectivity

## Abstract

Neuroimaging functional connectivity analyses have shown that the negative coupling between the amygdala and cortical regions is linked to better emotion regulation (ER) in experimental task settings. However, less is known about the neural correlates of ER traits or dispositions. The present study aimed to: (1) replicate the findings of differential cortico-limbic coupling during resting-state depending on the dispositional use of emotion regulation strategies. Furthermore, the study aimed to: (2) extend prior findings by examining whether differences in cortico-limbic coupling during resting-state predict experiential and neuronal ER success in a standard ER task. To this end, *N* = 107 healthy adults completed the Emotion Regulation Questionnaire (ERQ), underwent an 8-min resting-state fMRI acquisition, and completed a reappraisal task during fMRI. Functional connectivity maps of basolateral and centromedial amygdala nuclei were estimated with a seed-based approach regarding associations with regions of the prefrontal cortex and were then correlated with ERQ scores as well as experiential and neuronal ER success. All hypotheses and the analysis plan are preregistered at https://osf.io/8wsgu. Opposed to prior findings, we were not able to replicate a correlation of dispositional ER strategy use with functional connectivity between the amygdala and PFC regions (*p* > 0.05, FWE-corrected). Furthermore, there was no association of experiential and neuronal reappraisal success with functional connectivity between amygdala and insula as well as PFC (*p* > 0.05, FWE-corrected). The present preregistered study calls into question the reported association between individual differences in resting-state cortico-limbic connectivity and dispositional use of ER strategies. However, ongoing advances in functional brain imaging and distributed network approaches may leverage the identification of reliable functional connectivity patterns that underlie successful emotion regulation.

## Introduction

Emotion regulation (ER) is defined by the activation of a goal to change an unfolding emotional response and can be described as any process by which individuals modify their emotional experiences, expressions, and physiology (Gross, 1998, 2015). Being able to effectively regulate one’s emotional reactions is of crucial importance for appropriate social interactions and an essential feature of mental and physical health (Gross and Munoz, 1995; English et al., 2012; Kanske et al., 2012; Hu et al., 2014; Johnstone and Walter, 2014; Kret and Ploeger, 2015). The influential Process Model of Emotion Regulation and its extension (Gross, 1998, 2015) categorizes strategies of emotion regulation according to the time point in the emotion generation process, at which they are being implemented. Cognitive change (e.g., reappraisal) appears early in the process (antecedent-focused) and refers to altering the value of the emotion eliciting stimulus, whereas response modulation (e.g., expressive suppression) takes effect later and aims at altering the emotional response. The most studied reappraisal strategy is reinterpretation, which implies changing the meaning of a stimulus (Ochsner et al., 2002). Another reappraisal strategy is detachment (distancing) where one is taking the perspective of an uninvolved observer to reduce the subjective relevance of the stimuli (Kalisch et al., 2005; Walter et al., 2009). It has been assumed that cognitive reappraisal (reinterpretation and detachment) as antecedent-focused strategies are most effective because the emotional response has not fully unfolded and the negativity of an event itself is altered, whereas response-focused strategies such as expressive suppression often fail in fully modifying the emotional response since they are initiated later in the emotion-generative process (for a review see Gross, 2002). Nevertheless, people implement both strategies in their daily lives and results are pointing to expressive suppression being advantageous in some contexts (Bonanno et al., 2004; Bonanno and Burton, 2013), while reappraisal may also turn out unsuccessful (Aldao et al., 2015). Research that investigates the underlying mechanisms influencing these long and short-term outcomes of both strategies is still ongoing.

Until recently, this research has roughly followed two approaches (Tull and Aldao, 2015): A task-related, experimental approach (hereinafter referred to as task-related ER) and an approach investigating individual differences in ER abilities and dispositional use of strategies, respectively (hereinafter referred to as dispositional ER). The task-related approach uses experimental tasks, in which participants are instructed to use one or more ER strategies to decrease or increase (mostly negative) emotions and investigates effects on different emotional (experiential), behavioral and psychophysiological outcomes. The dispositional approach frequently relies on self-report questionnaires to evaluate ER abilities and dispositional use of ER strategies. One of the most widely used self-report measures of reappraisal (with an emphasis on reinterpretation) and expressive suppression is the Emotion Regulation Questionnaire (ERQ, Gross and John, 2003). Based on the Process Model of Emotion Regulation, the ERQ evaluates the dispositional use of these two strategies.

In experimental settings, task-related reappraisal is effective in changing emotional experiences, behavior, and physiological responses (Webb et al., 2012). The authors report detachment (*d*+ = 0.45) being significantly more advantageous than reinterpretation (*d*+ = 0.36). Expressive suppression proved to be effective in the regulation of emotional experiences and behavioral, but not physiological responses (Webb et al., 2012). In contrast, a recent meta-analysis on psychophysiological outcomes of task-related ER reports mixed findings with low to medium effect-sizes for both reappraisal and expressive suppression and mostly non-significant meta-analytical effects (Zaehringer et al., 2020). Concerning dispositional ER, the dispositional use of reappraisal, measured with the ERQ, has been linked to interpersonal functioning and psychological as well as physical well-being (Gross and John, 2003). Moreover, Aldao et al. (2010) could meta-analytically show that dispositional reappraisal is negatively associated with symptoms of psychopathology. In contrast, dispositional suppression was positively associated with psychopathology with medium to large effect sizes (Aldao et al., 2010; Hu et al., 2014), worse interpersonal functioning, and greater risk of depression (Gross and John, 2003). Hence, concerning emotional experiences, both strategies have shown to be successful in the short-term regulation of emotions, while there are mixed findings of short-term physiological outcomes. Reappraisal is advantageous concerning long-term (self-reported) psychological outcomes.

Neuroscientific studies of healthy but also impaired ER can contribute to the understanding of the mechanisms and underlying processes leading to these outcomes. Mostly, this research is investigating neuronal activity in brain regions implicated in cognitive control (i.e., the prefrontal cortex, PFC) and brain regions implicated in emotional processing (i.e., amygdala and insula) as well as the coupling between these structures. Functional brain imaging studies repeatedly showed that activation in the PFC [i.e., the anterior cingulate cortex (ACC), medial (m)PFC, and dorsolateral (dl) PFC] and reduction of amygdala activation is associated with a detachment in ER tasks (Kalisch et al., 2005; Walter et al., 2009; Erk et al., 2010; Koenigsberg et al., 2010; Schardt et al., 2010; Ochsner et al., 2012; Dörfel et al., 2014. During expressive suppression, activity within similar PFC regions and the supplementary motor area (SMA) has been reported (Phillips et al., 2008; Vrtička et al., 2011; Dörfel et al., 2014). In contrast, suppression has been associated with significant increases in the amygdala and insula activity (Goldin et al., 2008; Hayes et al., 2010; Vanderhasselt et al., 2013; Dörfel et al., 2014). Therefore, it can be assumed that the interaction between PFC regions and regions of emotional processing is different for the two strategies.

For successful ER, it is assumed that dorsal PFC regions exert an inhibitory effect on regions of emotional processing *via* ventral PFC regions (Ochsner et al., 2002; Wager et al., 2008; Lee et al., 2012; Buhle et al., 2014). Consequently, in reappraisal tasks, task-related functional connectivity has been reported between the amygdala and the PFC (Banks et al., 2007; Erk et al., 2010; Schardt et al., 2010; Winecoff et al., 2011; Sripada et al., 2014; Paschke et al., 2016). Moreover, the functional coupling of the amygdala with ventral and dorsal PFC regions were significantly correlated to experiential (self-reported) emotion regulation success (Banks et al., 2007; Paschke et al., 2016). Lee et al. (2012) suggested that functional coupling between the amygdala and prefrontal regions as well as pregenual ACC during cognitive reappraisal depends on individual differences in the capacity for reducing negative emotion. In line with this, studies including patients with psychological disorders with reduced ability to regulate emotions (e.g., depression and anxiety) have found deficits in functional and effective connectivity between the amygdala and frontal brain regions (Erk et al., 2010; Cullen et al., 2011; Niedtfeld et al., 2012; Clauss et al., 2014; Mochcovitch et al., 2014; Radaelli et al., 2015; Picó-Pérez et al., 2017). Additionally, there is evidence that not only task-related connectivity but also alterations in resting-state functional connectivity of (among others) PFC, amygdala, and insula are associated with depression and anxiety (Menon, 2011; Zhang et al., 2014; Barch, 2017). Resting-state functional brain connectivity (rsFC) reflects intrinsic connectivity, which is correlated temporal patterns among brain regions during rest. The resting-state networks closely match networks that have been continuously reported by different task conditions pointing to an intrinsic functional brain architecture important for task-specific brain activation (Smith et al., 2009). This also applies to ER-related brain networks (i.e., default mode network, the executive control network, and the salience network, see Beckmann et al., 2005; Damoiseaux et al., 2006; Seeley et al., 2007). Hence, it can be suggested that activity in task-related ER networks and resting-state connectivity between PFC and the amygdala show associations.

Gabard-Durnam et al. (2016) propose that experiences of stimulus-elicited coactivations in ER brain regions form these resting-state connectivity patterns (long-term phasic molding hypothesis), particularly during development in childhood and adolescence. The authors found that in a sample of children and adolescents, stimulus-elicited amygdala-mPFC connectivity predicted rsFC 2 years later. Likely, the dispositional, daily use of a specific ER strategy and the experience of (un)successful ER alters the functional architecture of these brain networks, which is represented in rsFC. In turn, it can be assumed that functional connectivity influences dispositional, daily strategy choice as well as strategy implementation.

Successful task-related ER (defined by a decrease in self-reported emotional experiences as well as a deactivation of brain regions engaged in emotion processing), as well as dispositional ER, should, therefore, be associated with rsFC between amygdala and PFC. However, few studies so far have directly investigated this. Picó-Pérez et al. (2018) found rsFC between the amygdala and PFC regions as well as the insula to be distinctly associated with dispositional use of suppression and reappraisal, respectively, as measured by the ERQ (Gross and John, 2003). In contrast, Uchida et al. (2015) could not find associations with self-reported dispositional ER (measured with the Difficulties in Emotion Regulation Scale, DERS, Gratz and Roemer, 2004). In a study by Morawetz et al. (2016), task-related ER (reinterpretation) success, defined by affect ratings, was positively correlated with rsFC between right amygdala and the left ventrolateral (vl)PFC as well as the insula. Uchida et al. (2015) demonstrated that greater reappraisal (reinterpretation) success, again measured by affect ratings, showed a significant negative correlation with rsFC of the right amygdala with mPFC. However, the two latter studies only focused on experiential ER success (affect ratings), and did not report results on neuronal ER success (defined by deactivations in regions of emotional processing). Additionally, to our knowledge, findings of these few existing studies have not been validated by replications.

Following this, the present study aimed at replicating and extending findings of associations between ER and rsFC of the amygdala and PFC. To define the replication attempts, we draw upon the definitions proposed by Zwaan et al. (2018), who differentiate between direct and conceptual replication studies. Direct replication is described as a study that attempts to recreate the critical elements (e.g., samples, procedures, and measures) of an original study. The authors underline that “a direct replication does not have to duplicate all aspects of an original study. Rather it must only duplicate those elements that are believed necessary for producing the original effect.” Conceptual replication is defined as a study with theoretically meaningful changes “to the original procedures that might make a difference concerning the observed effect size” (p. 3).

The present study specifically pursued three objectives: (1) we aimed at an investigation of whether individual differences in *dispositional reappraisal and expressive suppression* (defined by self-reported habitual use as measured with the ERQ) can explain variance in rsFC between amygdala and PFC. To do so, we reanalyzed own existing data (Diers et al., 2014; Scheffel et al., 2019) from three related ER experiments containing measures of dispositional use of reappraisal and suppression (*via* ERQ) and fMRI resting-state scans to replicate the findings by Picó-Pérez et al. (2018). We aimed at a direct replication according to the definition outlined above. Due to using existing data, there were methodological differences between our investigation and the Picó-Pérez et al.’s (2018) study which will be outlined in the “Materials and Methods” section and in Supplementary Table S23. However, these differences are mostly technical and do not lead to a different operationalization of the constructs.

(2) We aimed at investigating whether individual differences in task-related, *experiential reappraisal success* (as defined by a decrease in self-reported arousal during a reappraisal task) explain variance in rsFC between the amygdala and PFC. To this end, we reanalyzed existing data from the aforementioned experiments, which focused on detachment as a reappraisal strategy. This investigation is inspired by the study of Uchida et al. (2015). Because there are important differences concerning the operationalization of the constructs and the experimental procedure between our study and the Uchida study (see Supplementary Table S24), the current investigation can be a conceptual replication at best.

(3) Extending the findings of Uchida et al. (2015), we aimed at an investigation of associations between task-related, *neuronal reappraisal success* (as defined by a decrease of amygdala activity during emotion regulation) and rsFC between the amygdala and PFC, again using existing data of the aforementioned data sets.

### Replication Attempt and Extension of Existing Studies on rsFC and Dispositional as well as Task-Related Emotion Regulation Success

Picó-Pérez et al. (2018) reported that dispositional reappraisal was negatively correlated with rsFC between left basolateral amygdala and left insula as well as dACC, and between right basolateral amygdala and SMA/dACC as well as the left insula. For dispositional suppression, a positive correlation was found with rsFC between the right basolateral amygdala and dACC, a negative correlation with rsFC between the left centromedial amygdala and SMA. The study was conducted with 48 healthy participants (23 females) with a mean age of 39.7 years. Participants filled out the Spanish version of the ERQ and underwent a resting fMRI scan. Detailed methods in comparison to our replication attempts can be found in Supplementary Table S23.

Uchida et al. (2015) report a significant negative correlation of task-related experiential reappraisal success with rsFC between the right amygdala and mPFC. This study investigated 62 participants (32 females, mean age 22.3 years), reflecting a broad range of ER ability due to preselection according to DERS scores (Gratz and Roemer, 2004). The participants underwent a fMRI reappraisal task, where they were instructed to either attend to neutral or negative pictures or reinterpret the pictures to reduce their negative feelings (reinterpretation as reappraisal strategy). At the end of each trial, participants rated their negative emotional reactions (affect rating). Reappraisal success (reappraisal score) was defined as the difference between the affect rating for the Attend Negative condition minus the Reappraise Negative condition during scanning. Additionally, the participants underwent a resting fMRI scan. The authors did not report associations between rsFC and the fMRI responses during the reappraisal task (neuronal emotion regulation success). Detailed methods in comparison with our replication attempt can be found in Supplementary Table S24.

Based on the two studies described above, we developed the following hypotheses: Regarding aim 1, we took into account the connectivity results of Picó-Pérez et al. (2018) with and without global signal regression (GSR) and hypothesized that there is a significant negative correlation of *dispositional reappraisal use* with rsFC between (1a) left basolateral amygdala and left insula, (1b) left basolateral amygdala and dACC, (1c) right basolateral amygdala and left insula, (1d) right basolateral amygdala and the SMA/dACC. Additionally, we hypothesized that there is a negative correlation of *dispositional suppression use* with rsFC between (1e) left centromedial amygdala and the SMA, and (1f) right basolateral amygdala and dACC. Concerning aim 2, we took into account the results of both, Uchida et al. (2015) and Picó-Pérez et al. (2018), and hypothesized that there is a negative correlation of task-related *experiential reappraisal success* with rsFC between (2a) left amygdala and left insula, (2b) right amygdala and left insula, (2c) the amygdala and dorsomedial (dm) PFC, (2d) amygdala and ventromedial PFC, and (2e) a correlation of *experiential reappraisal success* with rsFC between the amygdala and dlPFC. Lastly, we hypothesized that there is a correlation between task-related *neuronal reappraisal success* with rsFC between (3a) the amygdala and insula, (3b) amygdala and dmPFC, and (3c) amygdala and dlPFC. Hypotheses, methods, and analysis plan were preregistered and can be found at https://osf.io/8wsgu.

## Materials and Methods

This study is a reanalysis of data collected within a larger project on neural correlates and individual differences of ER and its aftereffects (SFB 940 Project A5). To achieve a reliable sample size, we combined three samples from three slightly different ER experiments. Please note that results regarding research questions on task-related effects as well as associations with genetic polymorphisms are published elsewhere (Diers et al., in preparation; Gärtner et al., 2019; Scheffel et al., 2019). Results on the research question of this publication have not been reported in any of these publications. We report how we determined our sample size, all data exclusions (if any), all manipulations, and all measures in the study (Simmons et al., 2012). Data, scripts, and analysis routines can be found at https://osf.io/8wsgu.

Mostly due to the analysis of existing data, our procedures deviated from the methodological procedures of the original studies, differences can be found in detail in Supplementary Tables S23, S24.

### Participants

The sample size was defined based on feasibility considerations. This resulted in a target sample size of over 48 participants per experiment. At the end of the data collection, 136 healthy participants took part in the study, *N* = 42 in Experiment 1, *N* = 47 each in Experiment 2 and 3. Participants were mostly students from the local university community. All participants were right-handed, pre-screened for magnetic resonance imaging (MRI) contraindications (e.g., metal plates or implants), and had no current or prior medical, neurological, or psychiatric illness or treatment. The experimental protocol was approved by the ethics committee of the TU Dresden (EK 10012012). Participation was voluntary and written consent was obtained. Participants received financial compensation for their time and effort.

After inspection of the data, *N* = 29 had to be excluded because of missing resting-state sessions or due to missing significant parts of the amygdala in resting-state images. Data of *N* = 107 participants (64 female; age: 24.4 ± 4.2 years, range: 18–48) were analyzed (*N* = 27 in Experiment 1, *N* = 40 in Experiments 2 and 3, respectively). Please note that the sample size of some calculations is smaller due to missing questionnaire data or task-related fMRI data (see Table 1 below). Given our sample size, a power analysis with G*Power (Faul et al., 2009) indicated that for correlational analyses, we were able to detect an *r* of 0.31 with a power of 0.80 (two-tailed, α = 0.05/4 corrected for multiple comparisons for analyses on four amygdala nuclei).

**Table 1:** Sample characteristics and descriptive results of dispositional emotion regulation.

	*N*	M (±SD)
Age	106	24.4 (±4.2)
Gender (male/female)	106	42/64
ERQ reappraisal (α = 0.74)	101	4.8 (±0.8)
ERQ suppression (α = 0.76)	102	3.4 (±1.2)

*Note: α = Cronbach’s Alpha; differences in N are due to missing demographic or questionnaire data of single participants*.

### Study Procedure

All three experiments contained two sessions 1 week apart from each other. In the first session, a functional MRI (fMRI) measurement during an experimental emotion regulation task (ERT), self-reported arousal ratings (AR) after each experimental run, a structural MRI (sMRI) measurement, and a stimuli re-exposure fMRI run were performed. In the second session, a resting-state fMRI (RS-fMRI) and another stimuli re-exposure fMRI run were completed. Additionally, the participants filled in questionnaires measuring individual differences on several traits and abilities (see “Emotion Regulation (ER) Task and Experiential Reappraisal Success” section). Please refer to Supplementary Figure S1 for a detailed description of the experimental procedure.

### Emotion Regulation (ER) Task and Experiential Reappraisal Success

Participants performed an ER task with negative (categories: animal, body, disaster, disgust, injury, suffering, violence, and weapons) and neutral (categories: objects, persons, and scenes) pictures. Pictures were taken from the International Affective Picture System (IAPS, Lang et al., 2008) and the Emotional Picture Set (EmoPicS, Wessa et al., 2010). Pictures were divided into subsets and randomly assigned to conditions. Valence (V) and arousal (A) were comparable between the experiments: For negative pictures, values were *V* = 2.67–2.81 and *A* = 5.54–5.74 (Experiment 1), *V* = 2.65–2.71 and *A* = 5.69–5.85 (Experiment 2), and *V* = 2.65–2.71 and *A* = 5.55–5.85 (Experiment 3). For neutral pictures, values were *V* = 4.98–5.16 and *A* = 2.86–3.04 (Experiment 1), *V* = 5.13–5.17 and *A* = 2.94–2.96 (Experiment 2), and *V* = 5.13–5.19 and *A* = 2.85–2.96 (Experiment 3).

The ER tasks differed slightly across the three experiments. However, all had in common that participants went through a Permit and a Detach condition (Diers et al., 2014, in preparation; Gärtner et al., 2019; Scheffel et al., 2019). During the Permit condition, participants should take a close look at the pictures and permit any emotions, that might arise. During the Detach condition, they were asked to “take the position of a non-involved observer, thinking about the picture objectively.” Strategies were trained outside the MRI scanner.

Each experimental trial consisted of a stimulation period and a relaxation period. In the stimulation period, a picture was presented for 8 (Experiments 1 and 3), or 10 s (Experiment 2). Within the initial 2 s of this period, a semi-transparent overlay containing the instruction was presented in the center of the picture. Afterward, a fixation cross was presented (relaxation period). After each trial (Experiment 1) or block (Experiments 2 and 3), participants rated their emotional arousal. The difference between arousal ratings for the conditions Negative Permit and Negative Detach was determined as *experiential reappraisal success*.

After the ER experiment, participants were asked whether they followed the instructed strategies. All participants stated that they did so. For a more detailed description of the three experiments, please see Supplementary Methods.

### Psychometric Measurements (Dispositional Emotion Regulation and Affect)

Participants completed several questionnaires on personality traits, ER abilities, need for cognition, thought suppression, mindfulness, acceptance, worry, and anxiety (for a complete list of measures, see https://osf.io/8wsgu). The following questionnaires were used in the present study: The German version of the Emotion Regulation Questionnaire (ERQ; Gross and John, 2003; German version: Abler and Kessler, 2009) to determine *dispositional reappraisal and suppression use*, and the Positive and Negative Affect Schedule (PANAS, Watson et al., 1988; German version: Janke and Glöckner-Rist, 2014) to determine positive and negative affect.

### MRI Data Acquisition

Functional and structural imaging was performed on a 3.0 Tesla Siemens Magnetom Trio scanner (Siemens AG, Erlangen, Germany), using a 12-channel head coil. Functional data were obtained using a T2*-weighted echo-planar imaging sequence. The field of view (FOV) had a size of 192 × 192 mm^2^ with a matrix size of 64 × 64, flip angle 80°, slice gap 1 mm, repetition time (TR) = 2410 ms, and echo time (TE) = 25 ms. Forty-two axial slices were acquired with a voxel size of 3.0 × 3.0 × 2.0 mm^3^. Stimuli were presented using Presentation (Neurobehavioral Systems, Albany, CA, USA). For each subject, anatomical (T1-weighted) images were acquired using an MPRAGE sequence consisting of 176 sagittal slices with a thickness of 1 mm (TR: 1,900 ms, TE: 2.26 ms, flip angle 9°, FOV: 256 × 256 mm^2^, matrix size 256 × 256, voxel size: 1 × 1 × 1 mm^3^; Diers et al., 2014; Gärtner et al., 2019; Scheffel et al., 2019).

### Data Analysis

#### Resting-State Functional Connectivity

##### Seed and ROI Definition

**Figure 1:**
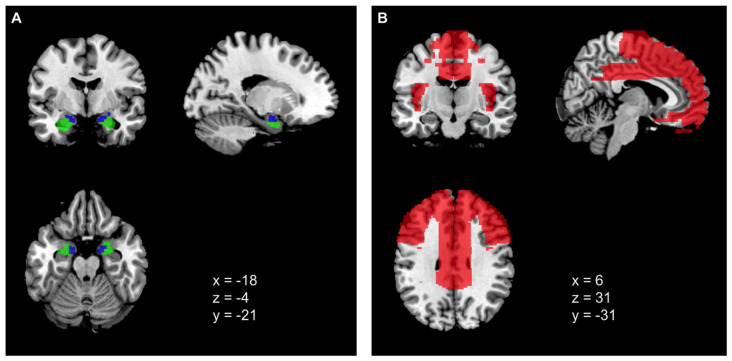
**(A)** Amygdala seed regions, blue = CMA, green = BLA. **(B)** Prefrontal cortex (PFC) ROI mask encompassing different regions of the frontal lobe, cingulate gyri, and insula (see Picó-Pérez et al., 2018).

The selection of the seed regions were based on Baur et al. (2013) and corresponded to left basolateral amygdala (BLA), right BLA, left centromedial amygdala (CMA), and right CMA for the resting-state analyses (see Figure 1A). For all four nuclei, maximum probability maps were created using the SPM Anatomy toolbox v.2.2c (Eickhoff et al., 2005). The probability threshold was set to 40% for each voxel to provide sufficient areal coverage of the anatomical structure (Eickhoff et al., 2006; Baur et al., 2013). Note that following Picó-Pérez et al. (2018), the CMA comprised centromedial and superficial divisions of the left and right amygdala.

The ROI mask for the PFC was restricted to a 56,833-voxel mask (2 × 2 × 2 mm^3^; see Figure 1B) created with the Wake Forest University (WFU) Pick-atlas toolbox (Maldjian et al., 2003). Following the procedure described by Picó-Pérez et al. (2018), the mask comprised different regions of the frontal lobe (i.e., inferior frontal, middle frontal, superior frontal, medial frontal and orbital gyri), the cingulate gyri and the insulae. Although we used the same regions, our ROI mask differed in size with the ROI mask by Picó-Pérez et al. (2018). Our contact with the authors did not solve the issue.

##### Data Preprocessing and Analysis

Preprocessing and statistical analyses of resting-state MRI data were carried out using the CONN toolbox (version 18b) pipeline (Whitfield-Gabrieli and Nieto-Castanon, 2012), SPM 12^1^ and Matlab 2019b (MathWorks, Natick, MA, USA). Preprocessing of the functional scans included spatial realignment and unwarping, slice-time correction, and outlier detection (ART-based scrubbing). Next, DARTEL (Ashburner, 2007) was used to create a study-specific anatomical template. Subject-specific normalization parameters were estimated for anatomical images. These parameters were then applied to the functional scans. Lastly, smoothing using an 8 mm Gaussian kernel was done. Before first-level analyses, a denoising procedure was applied to remove motion artifacts, physiological and other artifactual effects from the fMRI-signal. This procedure included the component-based correction method (Comp-Cor, Behzadi et al., 2007) and temporal band-pass filtering of 0.008–0.09 Hz. To avoid potential ramping effects at the beginning of the session, CONN models the entire acquisition and includes an additional confounding variable as a covariate in the denoising procedure. The six-movement parameters and a matrix containing the ART-detected outliers were included as first-level nuisance covariates. Preprocessing of the structural scans included segmentation and normalization to the MNI reference brain.

For first-level analysis, a general lineal model (GLM) was created which includes the four noise-corrected amygdala-seed time series as predictors. To check the whole brain, basic rsFC of the four amygdala nuclei were computed both for the whole sample as well as for the three experiments. All second-level analyses for hypotheses testing were restricted to the PFC mask. For second-level analysis of aim 1, separate multiple regression models were performed for each of the four amygdala seeds (left CMA, right CMA, left BLA, and right BLA). Dispositional reappraisal and suppression use served as predictors of interest to test for voxel-wise correlations between the seed-to-ROI connectivity values and ERQ subscales. For second-level analyses of aim 2, multiple regression models were performed for left and right amygdala seeds, respectively (each comprising the mean of both CMA and BLA nuclei) for hypotheses (2a) and (2b), and for each of the four amygdala seeds (*F*-Test for any effects among the four seeds) for hypotheses (2c) to (2e). Experiential reappraisal success (one predictor) served as a predictor of interest. For second-level analyses of aim 3, multiple regression models were performed for each of the four amygdala seeds (*F*-Test for any effects among the four seeds) for hypotheses (3a) to (3c). Neuronal reappraisal success (extracted mean activity from left BLA, right BLA, left CMA, right CMA during Negative Permit > Negative Detach, see “Task-Related Neuronal Reappraisal Success–Data Preprocessing and Statistical Analysis” section) served as predictors of interest, and the mean of all four predictors was computed during second-level contrast analysis. For all analyses, the number of Experiments (1, 2, 3) served as a covariate. The significance threshold was set to *p* < 0.05, family-wise error corrected (FWE) for multiple comparisons. For exploratory analyses, we lowered the threshold to *p* < 0.001 (uncorrected) and report respective results in the Supplementary Material.

##### Task-Related Neuronal Reappraisal Success—Data Preprocessing and Statistical Analysis

Preprocessing and statistical analyses of functional MRI data were carried out using SPM 8^2^, SPM 12^1^, and Matlab 2019b (MathWorks, Natick, MA, USA). The first four volumes of each run were discarded. Preprocessing included motion correction, coregistration of individual functional and anatomical images, spatial normalization (deviating from the preregistration) of the anatomical data to the MNI template, application of the estimated transformation parameters to the coregistered functional images using a resampling resolution of 2 × 2 × 2 mm^3^, and spatial smoothing of the functional images (FWHM 8 mm). For first-level analysis, a GLM was created with regressors based on experimental conditions (Experiment 1: View Neutral, View Negative, Permit Negative, Detach Negative; Experiment 2: Permit Neutral, Permit Negative, Detach Neutral, Detach Negative; Experiment 3: Permit Neutral, Permit Negative, Detach Neutral, Detach Negative, Intensify Neutral, Intensify Negative), as well as six additional motion regressors of no interest. Instructions and pictures were set together as one event. Temporal patterns were modeled as boxcar function (8 s duration (Experiment 1 and 3) and 10 s duration (Experiment 2), respectively) to cover sustained responses. All regressors were convolved with the canonical hemodynamic response function (HRF). All runs of the imaging experiments were combined within one fixed-effects model.

To obtain scores for *neuronal reappraisal success*, the mean activity of the amygdala for the contrast Negative Permit > Negative Detach was extracted for each participant using MarsBaR^3^. Therefore, maximum probability maps of the left BLA, right BLA, left CMA, and right CMA were created using the SPM Anatomy toolbox v.2.2c (Eickhoff et al., 2005). The probability threshold was set to 40% for each voxel to provide sufficient areal coverage of the anatomical structure (Eickhoff et al., 2006; Baur et al., 2013).

#### Dispositional ER and Task-Related Experiential Responses (Self-report)–Statistical Analysis

Analyses on task-related experiential responses (arousal ratings) and trait measures were conducted using R^4^. A Shapiro-Wilk test was performed to test variables for normal distribution. ERQ subscales (dispositional reappraisal and suppression use) were normally distributed (*p* > 0.05, see Supplementary Table S1). A paired *t*-test was conducted to check whether participants reported using reappraisal strategies to an equal extent than suppression strategies. The PANAS subscale positive affect and task-related experiential responses were not normally distributed (*p* < 0.05, see Supplementary Table S1). Wilcox signed-rank tests with continuity correction were conducted to check whether participants experienced positive and negative affect to an equal amount and to test whether task-related experiential responses were significantly lower after using detachment, compared to permitting all upcoming emotions.

## Results

### Dispositional ER and Task-Related Experiential Responses (Self-report)

The mean dispositional reappraisal and suppression scores (ERQ) are presented in Table 1 (for a comparison of all predictor variables across the three experiments see Supplementary Table S2). Participants reported using reappraisal (*M* = 4.8, *SD* = 0.8) to a significantly stronger extent than suppression (*M* = 3.4, *SD* = 1.2; *t*_(100)_ = 9.1, *p* < 0.001). Regarding positive and negative affect (PANAS), participants showed a significant higher experience of positive affect as compared to negative affect (*V* = 5241, *p* < 0.001). Correlation analyses showed a significant association between both PANAS subscales (*r* = −0.29, *p* = 0.003), but none between ERQ and PANAS subscales (*p* > 0.05).

In the ER experiment, participants reported significantly lower task-related experiential responses after detachment from negative pictures, *M* = −15.4, *SD* = 68.9, compared to permitting emotions, *M* = 13.6, *SD* = 59.8, *V* = 722.5, *p* < 0.001. Therefore, the implementation of the instructed ER strategies in the Experiment was successful (see Scheffel et al., 2019).

### Resting-State Functional Connectivity Results

#### Basic, Whole-Brain Functional Resting-State Connectivity (Without PFC Mask and Covariate)

Functional connectivity patterns of basolateral (BLA) and centromedial (CMA) amygdala seeds and regions within the whole brain for the whole sample without covariate are presented in Supplementary Figure S5. Overall, there were significant (*p* < 0.05 FWE-corrected) associations of left and right BLA and CMA nuclei with the amygdala, nucleus caudate, precentral and postcentral gyrus, Rolandic operculum, middle cingulum, angular gyrus, middle temporal gyrus, middle occipital gyrus, hippocampus, superior temporal pole and inferior parietal gyrus (see Supplementary Table S15 for a complete list of all associations).

Because of the differences between experiments (see Supplementary Table S2), we repeated all analyses separately for each experiment. The results are reported in Supplementary Figures S2 (Experiment 1), S3 (Experiment 2), S4 (Experiment 3), and Supplementary Tables S3 (Experiment 1), S7 (Experiment 2), S11 (Experiment 3).

#### Aim 1: Replication Dispositional Emotion Regulation and Functional Resting-State Connectivity

##### Whole Sample Results (With PFC Mask and Covariate)

Functional connectivity patterns of left and right basolateral (BLA) and centromedial (CMA) amygdala seeds and regions within the PFC mask are presented in Supplementary Table S19. Overall, there were no significant associations of left and right BLA and CMA with any region within the PFC mask (*p* > 0.05 FWE-corrected). For exploratory purposes, we lowered the threshold to *p* < 0.001 uncorrected. We found meaningful associations (clusters *k* ≥ 10 voxels) with superior orbitofrontal gyrus, SMA, inferior orbitofrontal gyrus, middle orbitofrontal gyrus, inferior frontal gyrus triangularis, superior frontal gyrus, insula, and Rolandic operculum.

Regarding dispositional emotion regulation, there were no significant correlations with rsFC of any of the four amygdala seeds, that is, neither dispositional reappraisal nor suppression use was positively or negatively correlated with rsFC between left and right BLA and CMA to any region within the PFC mask (*p* > 0.05 FWE-corrected). For exploratory purposes, we lowered the threshold to *p* < 0.001 uncorrected and found meaningful associations of dispositional emotion regulation (clusters *k* ≥ 10 voxels) with rsFC between brain regions: Reappraisal use was positively correlated with rsFC between right CMA and left insula, right BLA and right middle cingulum as well as left inferior frontal gyrus triangularis. Suppression use was positively correlated with rsFC between left CMA and right superior medial frontal gyrus, right inferior frontal gyrus opercularis, and left middle cingulum; and between right CMA and left middle frontal gyrus as well as right superior frontal gyrus. Furthermore, suppression scores were positively correlated with rsFC between left BLA and left superior temporal gyrus and right inferior frontal gyrus; and right BLA and right superior frontal gyrus. The results are presented in Table 2 and Supplementary Table S20.

**Table 2:** Significant clusters associated with the four amygdala nuclei as seeds restricted to PFC mask for reappraisal and suppression (aim 1).

Region	H	x	y	*z*	*k*	*T*	*p-*uncorr	*p*-FWE
*Reappraisal*								
*Left centromedial amygdala*								
No suprathreshold clusters								
*Right centromedial amygdala*								
Insula	L	−34	12	−14	49	4.16	<0.001	0.264
*Left basolateral amygdala*								
No suprathreshold clusters								
*Right basolateral amygdala*								
Middle Cingulum	R	4	−38	44	41	3.79	<0.001	0.602
Inferior Frontal Gyrus Triangularis	L	−54	38	20	10	3.64	<0.001	0.758
*Suppression*								
*Left centromedial amygdala*								
Superior Medial Frontal Gyrus	R	14	58	30	17	3.76	<0.001	0.645
Inferior Frontal Gyrus Opercularis	R	60	16	38	10	3.66	<0.001	0.744
Middle Cingulum	L	−8	−44	32	29	3.50	<0.001	0.882
*Right centromedial amygdala*								
Middle Frontal Gyrus	L	−30	20	44	34	4.30	<0.001	0.176
Superior Frontal Gyrus	R	18	26	40	14	3.75	<0.001	0.647
*Left basolateral amygdala*								
Superior Temporal Gyrus	L	−40	20	−16	11	3.67	<0.001	0.752
Inferior Frontal Gyrus Opercularis	R	56	16	36	15	3.50	<0.001	0.893
*Right basolateral amygdala*								
Superior Frontal Gyrus	R	18	24	40	23	4.29	<0.001	0.187

*Note. The significance threshold for seed-to-voxel analyses set at *p* < 0.001 uncorrected. Only clusters with *k* ≥ 10 are reported. Coordinates are given in MNI space. Amy, amygdala; R, right; L, left; H, Hemisphere*.

Because of differences between experiments (see Supplementary Table S2) we repeated all analyses separately for each experiment. The results are reported in the following.

##### Experiment 1

Regarding dispositional emotion regulation, there were no significant correlations with rsFC of any of the four amygdala seeds, that is, neither reappraisal nor suppression use was positively or negatively correlated with rsFC between left and right BLA and CMA to any region within the PFC mask (*p* > 0.05 FWE-corrected). For exploratory purposes, we lowered the threshold to *p* < 0.001 uncorrected. The results are reported in Supplementary Table S4.

##### Experiment 2

Regarding dispositional emotion regulation, there were no significant correlations with rsFC of any of the four amygdala seeds, that is, neither reappraisal nor suppression use was positively or negatively correlated with rsFC between left and right BLA and CMA to any region within the PFC mask (*p* > 0.05 FWE-corrected). For exploratory purposes, we lowered the threshold to *p* < 0.001 uncorrected. The results are reported in Supplementary Table S8.

##### Experiment 3

Regarding dispositional emotion regulation, there were no significant correlations with rsFC of any of the four amygdala seeds, that is, neither reappraisal nor suppression use was positively or negatively correlated with rsFC between left and right BLA and CMA to any region within the PFC mask (*p* > 0.05 FWE-corrected). For exploratory purposes, we lowered the threshold to *p* < 0.001 uncorrected. The results are reported in Supplementary Table S12.

#### Aim 2 and Aim 3: Extension to Experiential and Neuronal Reappraisal Success

The analyses were repeated with *experiential and neuronal reappraisal success*, respectively, as predictors of interest. Regarding experiential reappraisal success, there were no significant correlations with rsFC of the left and right amygdala to any region within the PFC mask (*p* > 0.05 FWE-corrected)^5^. For exploratory purposes, we lowered the threshold to *p* < 0.001 uncorrected and found meaningful associations (clusters *k* ≥ 10 voxels). Experiential reappraisal success was positively correlated with rsFC between left amygdala and left middle cingulum and left inferior frontal gyrus opercularis, and positively associated with rsFC between right amygdala and left middle cingulum (see Table 3 and Supplementary Table S21).

**Table 3:** Significant clusters associated with experiential reappraisal success with respective amygdala seeds restricted to PFC mask (aim 2).

Region	*H*	*x*	*y*	*z*	*k*	*T/F*	*p-*uncorr	*p*-FWE
*Left amygdala (BLA + CMA)*								
Middle Cingulum	L	−14	8	32	17	3.76	<0.001	0.663
Inferior Frontal Gyrus Opercularis	L	−38	10	12	19	3.67	<0.001	0.756
*Right amygdala (BLA + CMA)*								
Middle Cingulum	L	−12	14	32	21	3.88	<0.001	0.510
Precentral Gyrus	R	46	6	28	39	3.63	<0.001	0.765
*Amygdala (Any nucleus)*								
No suprathreshold clusters								

*Note. The significance threshold for seed-to-voxel analyses set at *p* < 0.001 uncorrected. Coordinates are given in MNI space. Amy, amygdala; R, right; L, left; H, Hemisphere; Only clusters with *k* ≥ 10 are reported*.

Regarding *neuronal reappraisal success*, there were no significant correlations with rsFC of the left and right amygdala to any region within the PFC mask (*p* > 0.05 FWE-corrected). For exploratory purposes, we lowered the threshold to *p* < 0.001 uncorrected. However, we found no meaningful associations (all clusters *k* < 10 voxels, except one association with superior frontal gyrus; see Table 4 and Supplementary Table S22).

**Table 4:** Significant clusters associated with neuronal reappraisal success for amygdala nuclei as seeds restricted to PFC mask (aim 3).

Region	H	x	y	*z*	*k*	*F*	*p-*uncorr	*p*-FWE
*Amygdala (Any nucleus)*								
Superior Frontal Gyrus	R	16	48	48	12	5.52	<0.001	0.894

*Note. The significance threshold for seed-to-voxel analyses set at *p* < 0.001 uncorrected. Coordinates are given in MNI space. Amy, amygdala; R, right; L, left; H, Hemisphere; Only clusters with *k* ≥ 10 are reported. Because of differences between experiments (see Supplementary Table S2) we repeated all analyses separately for each experiment. The results are reported in the following*.

##### Experiment 1

Regarding *experiential reappraisal success*, there were no significant correlations with rsFC of any of the four amygdala seeds, that is, changes in arousal ratings were not positively or negatively correlated with rsFC between left and right BLA and CMA to any region within the PFC mask (*p* > 0.05 FWE-corrected). For exploratory purposes, we lowered the threshold to *p* < 0.001 uncorrected. The results are reported in Supplementary Table S5.

Regarding *neuronal reappraisal success*, there were no significant correlations with rsFC of any of the four amygdala seeds, that is, mean activity of the amygdala for the contrast Negative Permit > Negative Detach was not positively or negatively correlated with rsFC between left and right BLA and CMA to any region within the PFC mask (*p* > 0.05 FWE-corrected). For exploratory purposes, we lowered the threshold to *p* < 0.001 uncorrected. The results are reported in Supplementary Table S6.

##### Experiment 2

Regarding *experiential reappraisal success*, there were no significant correlations with rsFC of any of the four amygdala seeds, that is, changes in arousal ratings were not positively or negatively correlated with rsFC between left and right BLA and CMA to any region within the PFC mask (*p* > 0.05 FWE-corrected). For exploratory purposes, we lowered the threshold to *p* < 0.001 uncorrected. The results are reported in Supplementary Table S9.

Regarding *neuronal reappraisal success*, there were no significant correlations with rsFC of any of the four amygdala seeds, that is, mean activity of the amygdala for the contrast Negative Permit > Negative Detach was not positively or negatively correlated with rsFC between left and right BLA and CMA to any region within the PFC mask (*p* > 0.05 FWE-corrected). For exploratory purposes, we lowered the threshold to *p* < 0.001 uncorrected. The results are reported in Supplementary Table S10.

##### Experiment 3

Regarding *experiential reappraisal success*, there were no significant correlations with rsFC of any of the four amygdala seeds, that is, changes in arousal ratings were not positively or negatively correlated with rsFC between left and right BLA and CMA to any region within the PFC mask (*p* > 0.05 FWE-corrected). For exploratory purposes, we lowered the threshold to *p* < 0.001 uncorrected. The results are reported in Supplementary Table S13.

Regarding *neuronal reappraisal success*, there were no significant correlations with rsFC of any of the four amygdala seeds, that is, mean activity of the amygdala for the contrast Negative Permit > Negative Detach was not positively or negatively correlated with rsFC between left and right BLA and CMA to any region within the PFC mask (*p* > 0.05 FWE-corrected). For exploratory purposes, we lowered the threshold to *p* < 0.001 uncorrected. The results are reported in Supplementary Table S14.

## Discussion

The first aim of this investigation was to directly replicate the study by Picó-Pérez et al. (2018). We analyzed associations of *dispositional emotion regulation* (ER), which is the habitual use of reappraisal and suppression measured *via* self-report (Abler and Kessler, 2009), with functional resting-state connectivity (rsFC) between the amygdalae and PFC by reanalyzing data from 107 participants of an ER study. None of the hypotheses could be confirmed, that is, we could not statistically confirm associations of dispositional reappraisal and suppression use with rsFC between left and right basolateral and centromedial amygdala, respectively, and regions in the PFC (ACC and SMA) and the insula. Thus, we failed to replicate the results of Picó-Pérez et al. (2018).

Second, we extended the investigation of resting-state functional networks and ER to associations with *experiential reappraisal success*. This investigation was based on findings by Uchida et al. (2015). Following the recommendations of Zwaan et al. (2018), we aimed at a conceptual replication and hypothesized that experiential reappraisal success (measured *via* arousal ratings) is associated with rsFC between left and right amygdala and left insula, with rsFC between left and right amygdala and dmPFC, with rsFC between the amygdalae and vmPFC, as well as with rsFC between the amygdala and dlPFC. Again, none of our hypotheses could be confirmed.

Lastly and to extend the research question to *neuronal reappraisal success*, we added a third analysis. Data of the same sample was analyzed to examine the hypotheses of associations between neuronal reappraisal success (defined by amygdala downregulation during reappraisal in an ER task) with rsFC between the amygdalae and insula, rsFC between the amygdalae and dmPFC, and rsFC between the amygdalae and dlPFC. We were not able to find any significant correlations here either.

To clarify whether there were any basic problems in detecting resting-state networks in our sample, we conducted a whole-brain functional connectivity analysis without any additional predictors and the covariate. This revealed that left and right basolateral and centromedial amygdala were negatively coupled with dlPFC, vlPFC, dmPFC, and dorsal ACC regions, and positively coupled with vmPFC, SMA, subgenual ACC as well as posterior cingulate gyrus and insula/vlPFC (see Supplementary Figure S5 and Supplementary Table S15). Overall, there is an overlap of these regions with the regions reported by Picó-Pérez et al. (2018), albeit not in the same direction. Similar connectivity maps have been reported by others (Roy et al., 2009; Weis et al., 2019 and Tetereva et al., 2020). We, therefore, assume that our resting state measurement has been successful in principle. However, when we included the number of Experiment as a covariate in this basic functional connectivity analysis, none of the clusters showed significant coupling with the amygdala anymore. Separate analyses of basic, whole-brain rsFC of the amygdala nuclei revealed variability between the three different sub-samples of our study (see Supplementary Figures S2–S4). However, this variability points to differences in strength rather than in the composition of the network.

There are several differences in methodology between the study of Picó-Pérez et al. (2018) and our study. Mainly, the differences refer to acquisition parameters of the functional MR images resulting, for instance, in a much lower spatial while slightly higher temporal resolution in Picó-Pérez et al. (2018). There were also differences in the preprocessing of fMRI data and statistical procedures (see Supplementary Table S23). Most importantly, we were not able to directly replicate the size of the PFC ROI for small volume correction. Although we followed the procedure laid out in the original study by Picó-Pérez et al. (2018), which resulted in an ROI of 17,391 voxels (2 × 2 × 2 mm^3^) in the original study, our mask contained 56,833 voxels (2 × 2 × 2 mm^3^). A visual comparison further points to some differences in coverage of the PFC, although the regions targeted by our hypotheses were included. Nevertheless, corrections for multiple comparisons had to be performed for a much smaller ROI in the Picó-Pérez study, which might have led to a higher possibility for smaller effects to reach statistical significance (see Figure 1). Moreover, differences are obvious regarding sample size and composition. The original study sample comprised 48 participants with a mean age of 39.6 years, while the participants in our study (*N* = 107) were much younger with a mean age of 24.4 years. Since emotion control, motives, as well as the choice of strategies change with age (e.g., Scheibe and Carstensen, 2010), this difference in mean age between the samples certainly plays a role. Because of the larger sample size, our study offers greater statistical power, which could have led to a reduced likelihood of false-positive findings.

Nevertheless, concerning aim 1, we did not achieve an exact but direct replication following Zwaan et al. (2018). A different definition of replications offers Brandt et al. (2014). They define close replications as studies that “aim to recreate a study as closely as possible so that ideally the only differences between the two are the inevitable ones” (p. 218). Concerning this strict definition, we did not achieve a close replication of the Pidó-Pérez study. However, we do not consider the differences in data acquisition and preprocessing to produce the failed replication, but the differences between the samples might at least partly explain the divergent results. However, we think that a replication of the same results should be rather independent of the detailed methodology.

Concerning aim 2, the methodological differences between our study and Uchida et al. (2015) are more pronounced. While the samples’ mean age is very similar (see Supplementary Table S24), the sample size is larger in our study. Additionally, Uchida et al. (2015) selected their participants according to their ER abilities to achieve an equal distribution of their abilities. This was not the case in our study. Thus, the original study ensured a higher variability of their main construct, which might have increased the possibility of finding an effect. Concerning the operationalization of experiential reappraisal success, the original study instructed reinterpretation as ER strategy and measured trial-by-trial affect ratings (Uchida et al., 2015), whereas in our replication attempt distancing was used as ER strategy and trial-by-trial ratings were only implemented in one of our data sets. Additionally, all of our experiments used arousal ratings. With these different operationalizations of a central construct, our replication attempt could be considered as conceptual (Zwaan et al., 2018) at best. In other words, “on a continuum from ‘close’ to ‘conceptual’ ” (Brandt et al., 2014) our replication attempt might be placed at the very end of the continuum. Thus, we can only conclude that the findings of the original study could not conceptually be replicated, the results do not extend to a different reappraisal strategy nor arousal instead of affect outcomes. Minor differences between the original and the replication can be found in data acquisition and processing, however, we consider these negligible (see Supplementary Tables S23, S24 for a detailed comparison).

Our findings not only contrast with the two studies on which we based our *a priori* hypotheses (Uchida et al., 2015; Picó-Pérez et al., 2018), they also contradict several other studies that have identified patterns of intrinsic functional connectivity that differ between the dispositional use of ER strategies or are associated with experiential and neuronal reappraisal success (Morawetz et al., 2016; Pan et al., 2018; Burr et al., 2020). Common to these studies is that regions in the default mode network have been identified. Particularly, the latest study by Burr et al. (2020) used the largest sample up to date (*N* = 1,316) in a data-driven, theory-free approach and found that intrinsic connectivity of the default mode network was associated with dispositional use of suppression (but not reappraisal). Critically, the authors used general functional connectivity (GFC, Elliott et al., 2019) to leverage shared features of task and resting-state fMRI and circumvent reported reliability issues of resting-state measures (e.g., Noble et al., 2019). Thus, instead of focusing on connectivity in *a priori* regions of interest between cortical and subcortical areas, distributed networks of brain regions might be a more promising target in future studies as they take into account the complexity of the underlying neuronal processes.

### Limitations

Several limitations have to be noted. First, to achieve a larger sample size and power, we combined three samples from three slightly different ER experiments. Although all experiments included the conditions and instructions relevant for the present study, subtle effects of experimental variation cannot be ruled out (e.g., Experiment 3 included an intensify instruction that was not present in Experiments 1 and 2). Therefore, we included Experiment, as a covariate in all our analyses (see also Supplementary Table S2 for a comparison of all predictor variables across experiments). Related to the study design, the resting-state measurement took place in a separate session approximately 1 week after the ER task. Although the investigated effects are supposed to be independent of each other (resting-state vs. task-related), unknown effects of time cannot be excluded since the experimental protocol was not randomized.

Second, the results regarding experiential reappraisal success are limited, because in Experiments 2 and 3 arousal ratings were recorded retrospectively after each block. In the overall research question of the larger study we were interested in aftereffects of ER (see for instance Walter et al., 2009). However, an arousal rating after picture offset in the relaxation period would alter time courses of the HRF (Burklund et al., 2014), thus, no trial-by-trial arousal rating was used while accepting the disadvantages of a retrospective arousal rating. Supplementary Table S2 presents the arousal ratings separately for each experiment. Indeed, the arousal ratings were higher for the trial-by-trial rating in Experiment 1 compared to the retrospective ratings in Experiments 2 and 3.

Third, the fixation of presented pictures was not controlled for *via* eye-tracking. Therefore, we do not have an objective measure to assess whether participants fixated negative images as instructed. This could have led to a failed activation of brain regions related to emotional processing during the negative stimulation period and, subsequently, to difficulties in detecting a reappraisal success. However, analyses of brain activation during reappraisal of negative pictures as compared to viewing negative pictures revealed downregulation of amygdala activation as well as activation in prefrontal regions in a previous analysis of the same data (Scheffel et al., 2019) replicating earlier findings (Walter et al., 2009; Buhle et al., 2014; Dörfel et al., 2014; Paschke et al., 2016).

Finally, we have no information on whether detachment is the participants’ preferred ER (reappraisal) strategy. Some participants may use other forms of reappraisal in their everyday life, for example, reinterpretation. While performing the task, they might be more successful with their preferred instead of the instructed strategy. However, we tried to address this by a training session on the implementation of detachment before the scanner session.

## Conclusion

In conclusion, the present replication study calls into question the reported findings on individual differences in resting-state cortico-limbic functional connectivity related to dispositional use of ER strategies and even task-related, experiential, and neuronal reappraisal success. The most parsimonious explanation for the lack of replication is that these differences are either small or non-existent, and/or swamped by sample effects and methodological differences. However, we remain optimistic that continued developments towards improving methodology in resting-state measurement (enhancing reliability) and distributed network approaches will help to eventually reveal reliable patterns of functional connectivity underlying successful emotion regulation.

## Data Availability Statement

The datasets generated and analyzed during this study can be found at the Open Science Framework (https://osf.io/p7hb5/).

## Ethics Statement

The studies involving human participants were reviewed and approved by Ethics committee of the TU Dresden (EK 10012012). The patients/participants provided their written informed consent to participate in this study.

## Author Contributions

AG and DD contributed to the design of the study. AG and CS organized the database and performed the statistical analysis. DD, CS, and AG wrote the first draft of the manuscript. All authors contributed to manuscript revision, read and approved the submitted version.

## Conflict of Interest

The authors declare that the research was conducted in the absence of any commercial or financial relationships that could be construed as a potential conflict of interest.

## Footnotes

^1^https://www.fil.ion.ucl.ac.uk/spm/software/spm12/

^2^https://www.fil.ion.ucl.ac.uk/spm/software/spm8/

^3^http://marsbar.sourceforge.net/

^4^https://www.r-project.org/

^5^Because values for experiential reappraisal success differed significantly between the three experiments (see Supplementary Table S2), we repeated the analyses with *z*-standardized values for this variable. However, there were still no significant correlations with rsFC of left and right amygdala to any region within the PFC mask (*p* > 0.05 FWE-corrected).

## References

[B1] Abler B. Kessler H. (2009). Emotion regulation questionnaire—A German version of the ERQ by Gross and John. Diagnostica 55, 144–152. 10.1026/0012-1924.55.3.144

[B2] Aldao A. Nolen-Hoeksema S. Schweizer S. (2010). Emotion-regulation strategies across psychopathology: a meta-analytic review. Clin. Psychol. Rev. 30, 217–237. 10.1016/j.cpr.2009.11.004 20015584

[B3] Aldao A. Sheppes G. Gross J. J. (2015). Emotion regulation flexibility. Cogn. Ther. Res. 39, 263–278. 10.1007/s10608-014-9662-4

[B4] Ashburner J. (2007). A fast diffeomorphic image registration algorithm. NeuroImage 38, 95–113. 10.1016/j.neuroimage.2007.07.007 17761438

[B5] Banks S. J. Eddy K. T. Angstadt M. Nathan P. J. Phan K. L. (2007). Amygdala-frontal connectivity during emotion regulation. Soc. Cogn. Affect. Neurosci. 2, 303–312. 10.1093/scan/nsm029 18985136 PMC2566753

[B6] Barch D. M. (2017). Resting-state functional connectivity in the human connectome project: current status and relevance to understanding psychopathology. Harv. Rev. Psychiatry 25, 209–217. 10.1097/hrp.0000000000000166 28816791 PMC5644502

[B7] Baur V. Hänggi J. Langer N. Jäncke L. (2013). Resting-state functional and structural connectivity within an insula-amygdala route specifically index state and trait anxiety. Biol. Psychiatry 73, 85–92. 10.1016/j.biopsych.2012.06.003 22770651

[B8] Beckmann C. F. DeLuca M. Devlin J. T. Smith S. M. (2005). Investigations into resting-state connectivity using independent component analysis. Philos. Trans. R. Soc. B Biol. Sci. 360, 1001–1013. 10.1098/rstb.2005.1634 16087444 PMC1854918

[B9] Behzadi Y. Restom K. Liau J. Liu T. T. (2007). A component based noise correction method (CompCor) for BOLD and perfusion based fMRI. NeuroImage 37, 90–101. 10.1016/j.neuroimage.2007.04.042 17560126 PMC2214855

[B10] Bonanno G. A. Burton C. L. (2013). Regulatory flexibility: an individual differences perspective on coping and emotion regulation. Perspect. Psychol. Sci. 8, 591–612. 10.1177/1745691613504116 26173226

[B11] Bonanno G. A. Papa A. Lalande K. Westphal M. Coifman K. (2004). The importance of being flexible: the ability to both enhance and suppress emotional expression predicts long-term adjustment. Psychol. Sci. 15, 482–487. 10.1111/j.0956-7976.2004.00705.x 15200633

[B12] Brandt M. J. Ijzerman H. Dijksterhuis A. Farach F. J. Geller J. Giner-Sorolla R. (2014). The replication recipe: what makes for a convincing replication’. J. Exp. Soc. Psychol. 50, 217–224. 10.1016/j.jesp.2013.10.005

[B13] Buhle J. T. Silvers J. A. Wager T. D. Lopez R. Onyemekwu C. Kober H.. (2014). Cognitive reappraisal of emotion: a meta-analysis of human neuroimaging studies. Cereb. Cortex 24, 2981–2990. 10.1093/cercor/bht154 23765157 PMC4193464

[B14] Burklund L. J. Creswell J. D. Irwin M. R. Lieberman M. D. (2014). The common and distinct neural bases of affect labeling and reappraisal in healthy adults. Front. Psychol. 5:221. 10.3389/fpsyg.2014.00221 24715880 PMC3970015

[B15] Burr D. A. d’Arbeloff T. Elliott M. L. Knodt A. R. Brigidi B. D. Hariri A. R. (2020). Functional connectivity predicts the dispositional use of expressive suppression but not cognitive reappraisal. Brain Behav. 10:e01493. 10.1002/brb3.1493 31930667 PMC7010583

[B16] Clauss J. A. Avery S. N. VanDerKlok R. M. Rogers B. P. Cowan R. L. Benningfield M. M.. (2014). Neurocircuitry underlying risk and resilience to social anxiety disorder. Depress Anxiety 31, 822–833. 10.1002/da.22265 24753211 PMC4314099

[B17] Cullen K. R. Vizueta N. Thomas K. M. Han G. J. Lim K. O. Camchong J.. (2011). Amygdala functional connectivity in young women with borderline personality disorder. Brain Connect. 1, 61–71. 10.1089/brain.2010.0001 22432955 PMC3621316

[B18] Damoiseaux J. S. Rombouts S. A. R. B. Barkhof F. Scheltens P. Stam C. J. Smith S. M.. (2006). Consistent resting-state networks across healthy subjects. Proc. Natl. Acad. Sci. U S A 103, 13848–13853. 10.1073/pnas.0601417103 16945915 PMC1564249

[B20] Diers K. Weber F. Brocke B. Strobel A. Schonfeld S. (2014). Instructions matter: a comparison of baseline conditions for cognitive emotion regulation paradigms. Front. Psychol. 5:347. 10.3389/fpsyg.2014.00347 24808872 PMC4009445

[B21] Dörfel D. Lamke J. P. Hummel F. Wagner U. Erk S. Walter H. (2014). Common and differential neural networks of emotion regulation by detachment, reinterpretation, distraction and expressive suppression: a comparative fmri investigation. NeuroImage 101, 298–309. 10.1016/j.neuroimage.2014.06.051 24993897

[B22] Eickhoff S. B. Heim S. Zilles K. Amunts K. (2006). Testing anatomically specified hypotheses in functional imaging using cytoarchitectonic maps. NeuroImage 32, 570–582. 10.1016/j.neuroimage.2006.04.204 16781166

[B23] Eickhoff S. B. Stephan K. E. Mohlberg H. Grefkes C. Fink G. R. Amunts K.. (2005). A new SPM toolbox for combining probabilistic cytoarchitectonic maps and functional imaging data. NeuroImage 25, 1325–1335. 10.1016/j.neuroimage.2004.12.034 15850749

[B24] Elliott M. L. Knodt A. R. Cooke M. Kim M. J. Melzer T. R. Keenan R.. (2019). General functional connectivity: shared features of resting-state and task fMRI drive reliable and heritable individual differences in functional brain networks. NeuroImage 189, 516–532. 10.1016/j.neuroimage.2019.01.068 30708106 PMC6462481

[B25] English T. John O. P. Srivastava S. Gross J. J. (2012). Emotion regulation and peer-rated social functioning: a 4-year longitudinal study. J. Res. Pers. 46, 780–784. 10.1016/j.jrp.2012.09.006 23471162 PMC3587109

[B26] Erk S. Mikschl A. Stier S. Ciaramidaro A. Gapp V. Weber B.. (2010). Acute and sustained effects of cognitive emotion regulation in major depression. J. Neurosci. 30, 15726–15734. 10.1523/JNEUROSCI.1856-10.2010 21106812 PMC6633759

[B28] Faul F. Erdfelder E. Buchner A. Lang A. G. (2009). Statistical power analyses using G*Power 3.1: tests for correlation and regression analyses. Behav. Res. Methods 41, 1149–1160. 10.3758/brm.41.4.1149 19897823

[B29] Gabard-Durnam L. J. Gee D. G. Goff B. Flannery J. Telzer E. Humphreys K. L.. (2016). Stimulus-elicited connectivity influences resting- state connectivity years later in human development: a prospective study. J. Neurosci. 36, 4771–4784. 10.1523/JNEUROSCI.0598-16.2016 27122035 PMC4846673

[B30] Gärtner A. Dörfel D. Diers K. Witt S. H. Strobel A. Brocke B. (2019). Impact of FAAH genetic variation on fronto-amygdala function during emotional processing. Eur. Arch. Psychiatry Clin. Neurosci. 269, 209–221. 10.1007/s00406-018-0944-9 30291441

[B31] Goldin P. R. McRae K. Ramel W. Gross J. J. (2008). The neural bases of emotion regulation: reappraisal and suppression of negative emotion. Biol. Psychiatry 63, 577–586. 10.1016/j.biopsych.2007.05.031 17888411 PMC2483789

[B32] Gratz K. L. Roemer L. (2004). Multidimensional assessment of emotion regulation and dysregulation: development, factor structure, and initial validation of the difficulties in emotion regulation scale. J. Psychopathol. Behav. Assess. 26, 41–54. 10.1023/b:joba.0000007455.08539.94

[B33] Gross J. J. (1998). Antecedent- and response-focused emotion regulation: divergent consequences for experience, expression, and physiology. J. Pers. Soc. Psychol. 74, 224–237. 10.1037/0022-3514.74.1.224 9457784

[B34] Gross J. J. (2002). Emotion regulation: affective, cognitive, and social consequences. Psychophysiology 39, 281–291. 10.1017/s0048577201393198 12212647

[B35] Gross J. J. (2015). The extended process model of emotion regulation: elaborations, applications, and future directions REPLY. Psychol. Inq. 26, 130–137. 10.1080/1047840x.2015.989751

[B36] Gross J. J. John O. P. (2003). Individual differences in two emotion regulation processes: implications for affect, relationships, and well-being. J. Pers. Soc. Psychol. 85, 348–362. 10.1037/0022-3514.85.2.348 12916575

[B37] Gross J. J. Munoz R. F. (1995). Emotion regulation and mental-health. Clin. Psychol. Sci. Pract. 2, 151–164.

[B38] Hayes J. P. Morey R. A. Petty C. M. Seth S. Smoski M. J. McCarthy G.. (2010). Staying cool when things get hot: emotion regulation modulates neural mechanisms of memory encoding. Front. Hum. Neurosci. 4:230. 10.3389/fnhum.2010.00230 21212840 PMC3015134

[B39] Hu T. Zhang D. Wang J. Mistry R. Ran G. Wang X. (2014). Relation between emotion regulation and mental health: a meta-analysis review. Psychol. Rep. 114, 341–362. 10.2466/03.20.pr0.114k22w4 24897894

[B40] Janke S. Glöckner-Rist A. (2014). Deutsche version der positive and negative affect schedule (PANAS). Zusammenstellung sozialwissenschaftlicher Items und Skalen (ZIS). 10.6102/zis146

[B41] Johnstone T. Walter H. (2014). “The neural basis of emotion dysregulation,” in Handbook of Emotion Regulation, 2nd edition, ed. Gross J. J. (New York, NY: The Guilford Press), 58–75.

[B42] Kalisch R. Wiech K. Critchley H. D. Seymour B. O’Doherty J. P. Oakley D. A.. (2005). Anxiety reduction through detachment: subjective, physiological, and neural effects. J. Cogn. Neurosci. 17, 874–883. 10.1162/0898929054021184 15969906

[B43] Kanske P. Heissler J. Schönfelder S. Wessa M. (2012). Neural correlates of emotion regulation deficits in remitted depression: the influence of regulation strategy, habitual regulation use and emotional valence. NeuroImage 61, 686–693. 10.1016/j.neuroimage.2012.03.089 22613776

[B44] Koenigsberg H. W. Fan J. Ochsner K. N. Liu X. Guise K. Pizzarello S.. (2010). Neural correlates of using distancing to regulate emotional responses to social situations. Neuropsychologia 48, 1813–1822. 10.1016/j.neuropsychologia.2010.03.002 20226799 PMC2905649

[B45] Kret M. E. Ploeger A. (2015). Emotion processing deficits: a liability spectrum providing insight into comorbidity of mental disorders. Neurosci. Biobehav. Rev. 52, 153–171. 10.1016/j.neubiorev.2015.02.011 25725415

[B46] Lang P. J. Bradley M. M. Cuthbert B. N. (2008). International Affective Picture System (IAPS): Affective Ratings of Pictures and Instruction Manual. Gainsville, FL: University of Florida.

[B47] Lee H. Heller A. S. van Reekum C. M. Nelson B. Davidson R. J. (2012). Amygdala-prefrontal coupling underlies individual differences in emotion regulation. NeuroImage 62, 1575–1581. 10.1016/j.neuroimage.2012.05.044 22634856 PMC3408571

[B48] Maldjian J. A. Laurienti P. J. Kraft R. A. Burdette J. H. (2003). An automated method for neuroanatomic and cytoarchitectonic atlas-based interrogation of fMRI data sets. NeuroImage 19, 1233–1239. 10.1016/s1053-8119(03)00169-1 12880848

[B49] Menon V. (2011). Large-scale brain networks and psychopathology: a unifying triple network model. Trends Cogn. Sci. 15, 483–506. 10.1016/j.tics.2011.08.003 21908230

[B50] Mochcovitch M. D. da Rocha Freire R. C. Garcia R. F. Nardi A. E. (2014). A systematic review of fMRI studies in generalized anxiety disorder: evaluating its neural and cognitive basis. J. Affect. Disord. 167, 336–342. 10.1016/j.jad.2014.06.041 25020268

[B51] Morawetz C. Kellermann T. Kogler L. Radke S. Blechert J. Derntl B. (2016). Intrinsic functional connectivity underlying successful emotion regulation of angry faces. Soc. Cogn. Affect. Neurosci. 11, 1980–1991. 10.1093/scan/nsw107 27510495 PMC5141959

[B52] Niedtfeld I. Kirsch P. Schulze L. Herpertz S. C. Bohus M. Schmahl C. (2012). Functional connectivity of pain-mediated affect regulation in borderline personality disorder. PLoS One 7:e33293. 10.1371/journal.pone.0033293 22428013 PMC3299768

[B53] Noble S. Scheinost D. Constable R. T. (2019). A decade of test-retest reliability of functional connectivity: a systematic review and meta-analysis. NeuroImage 203:116157. 10.1016/j.neuroimage.2019.116157 31494250 PMC6907736

[B54] Ochsner K. N. Bunge S. A. Gross J. J. Gabrieli J. D. (2002). Rethinking feelings: an FMRI study of the cognitive regulation of emotion. J. Cogn. Neurosci. 14, 1215–1229. 10.1162/089892902760807212 12495527

[B55] Ochsner K. N. Silvers J. A. Buhle J. T. (2012). Functional imaging studies of emotion regulation: a synthetic review and evolving model of the cognitive control of emotion. Ann. N Y Acad. Sci. 1251, E1–24. 10.1111/j.1749-6632.2012.06751.x 23025352 PMC4133790

[B56] Pan J. Zhan L. Hu C. Yang J. Wang C. Gu L.. (2018). Emotion regulation and complex brain networks: association between expressive suppression and efficiency in the fronto-parietal network and default-mode network. Front. Hum. Neurosci. 12:70. 10.3389/fnhum.2018.00070 29662443 PMC5890121

[B57] Paschke L. M. Dörfel D. Steimke R. Trempler I. Magrabi A. Ludwig V. U.. (2016). Individual differences in self-reported self-control predict successful emotion regulation. Soc. Cogn. Affect. Neurosci. 11, 1193–1204. 10.1093/scan/nsw036 27013102 PMC4967798

[B58] Phillips M. L. Ladouceur C. D. Drevets W. C. (2008). A neural model of voluntary and automatic emotion regulation: implications for understanding the pathophysiology and neurodevelopment of bipolar disorder. Mol. Psychiatry 13, 829, 833–857. 10.1038/mp.2008.65 18574483 PMC2745893

[B59] Picó-Pérez M. Alonso P. Contreras-Rodriguez O. Martinez-Zalacain I. Lopez-Sola C. Jimenez-Murcia S.. (2018). Dispositional use of emotion regulation strategies and resting-state cortico-limbic functional connectivity. Brain Imaging Behav. 12, 1022–1031. 10.1007/s11682-017-9762-3 28866781

[B60] Picó-Pérez M. Radua J. Steward T. Menchon J. M. Soriano-Mas C. (2017). Emotion regulation in mood and anxiety disorders: a meta-analysis of fMRI cognitive reappraisal studies. Prog. Neuropsychopharmacol. Biol. Psychiatry 79, 96–104. 10.1016/j.pnpbp.2017.06.001 28579400

[B61] Radaelli D. Sferrazza Papa G. Vai B. Poletti S. Smeraldi E. Colombo C.. (2015). Fronto-limbic disconnection in bipolar disorder. Eur. Psychiatry 30, 82–88. 10.1016/j.eurpsy.2014.04.001 24853295

[B62] Roy A. K. Shehzad Z. Margulies D. S. Kelly A. M. Uddin L. Q. Gotimer K.. (2009). Functional connectivity of the human amygdala using resting state fMRI. NeuroImage 45, 614–626. 10.1016/j.neuroimage.2008.11.030 19110061 PMC2735022

[B63] Schardt D. M. Erk S. Nusser C. Nothen M. M. Cichon S. Rietschel M.. (2010). Volition diminishes genetically mediated amygdala hyperreactivity. NeuroImage 53, 943–951. 10.1016/j.neuroimage.2009.11.078 19969089

[B64] Scheffel C. Diers K. Schönfeld S. Brocke B. Strobel A. Dörfel D. (2019). Cognitive emotion regulation and personality: an analysis of individual differences in the neural and behavioral correlates of successful reappraisal. Personal. Neurosci. 2:e11. 10.1017/pen.2019.11 32435746 PMC7219681

[B65] Scheibe S. Carstensen L. L. (2010). Emotional aging: recent findings and future trends. J. Gerontol. B Psychol. Sci. Soc. Sci. 65B, 135–144. 10.1093/geronb/gbp132 20054013 PMC2821944

[B66] Seeley W. W. Menon V. Schatzberg A. F. Keller J. Glover G. H. Kenna H.. (2007). Dissociable intrinsic connectivity networks for salience processing and executive control. J. Neurosci. 27, 2349–2356. 10.1523/jneurosci.5587-06.2007 17329432 PMC2680293

[B67] Simmons J. P. Nelson L. D. Simonsohn U. (2012). A 21 word solution. Available online at: https://ssrn.com/abstract=2160588 10.2139/ssrn.2160588. Accessed October 14, 2012

[B68] Smith S. M. Fox P. T. Miller K. L. Glahn D. C. Fox P. M. Mackay C. E.. (2009). Correspondence of the brain’s functional architecture during activation and rest. Proc. Natl. Acad. Sci. U S A 106, 13040–13045. 10.1073/pnas.0905267106 19620724 PMC2722273

[B69] Sripada C. Angstadt M. Kessler D. Phan K. L. Liberzon I. Evans G. W.. (2014). Volitional regulation of emotions produces distributed alterations in connectivity between visual, attention control and default networks. NeuroImage 89, 110–121. 10.1016/j.neuroimage.2013.11.006 24246489 PMC3955705

[B70] Tetereva A. O. Balaev V. V. Kartashov S. I. Ushakov V. L. Ivanitsky A. M. Martynova O. V. (2020). Asymmetry of amygdala resting-state functional connectivity in healthy human brain. Neuroreport 31, 17–21. 10.1097/wnr.0000000000001353 31651703

[B71] Tull M. T. Aldao A. (2015). Editorial overview: new directions in the science of emotion regulation. Curr. Opin. Psychol. 3, IV–X. 10.1016/j.copsyc.2015.03.009

[B72] Uchida M. Biederman J. Gabrieli J. D. E. Micco J. de Los Angeles C. Brown A.. (2015). Emotion regulation ability varies in relation to intrinsic functional brain architecture. Soc. Cogn. Affect. Neurosci. 10, 1738–1748. 10.1093/scan/nsv059 25999363 PMC4666109

[B73] Vanderhasselt M. A. Kühn S. De Raedt R. (2013). ‘Put on your poker face’: neural systems supporting the anticipation for expressive suppression and cognitive reappraisal. Soc. Cogn. Affect. Neurosci. 8, 903–910. 10.1093/scan/nss090 22956675 PMC3831557

[B74] Vrtička P. Sander D. Vuilleumier P. (2011). Effects of emotion regulation strategy on brain responses to the valence and social content of visual scenes. Neuropsychologia 49, 1067–1082. 10.1016/j.neuropsychologia.2011.02.020 21345342

[B75] Wager T. D. Davidson M. L. Hughes B. L. Lindquist M. A. Ochsner K. N. (2008). Prefrontal-subcortical pathways mediating successful emotion regulation. Neuron 59, 1037–1050. 10.1016/j.neuron.2008.09.006 18817740 PMC2742320

[B76] Walter H. von Kalckreuth A. Schardt D. Stephan A. Goschke T. Erk S. (2009). The temporal dynamics of voluntary emotion regulation. PLoS One 4:e6726. 10.1371/journal.pone.0006726 21949675 PMC3175755

[B77] Watson D. Clark L. A. Tellegen A. (1988). Development and validation of brief measures of positive and negative affect: the PANAS scales. J. Pers. Soc. Psychol. 54, 1063–1070. 10.1037/0022-3514.54.6.1063 3397865

[B78] Webb T. L. Miles E. Sheeran P. (2012). Dealing with feeling: a meta-analysis of the effectiveness of strategies derived from the process model of emotion regulation. Psychol. Bull. 138, 775–808. 10.1037/a0027600 22582737

[B79] Weis C. N. Huggins A. A. Bennett K. P. Parisi E. A. Larson C. L. (2019). High-resolution resting-state functional connectivity of the extended amygdala. Brain Connect. 9, 627–637. 10.1089/brain.2019.0688 31389253

[B80] Wessa M. Kanske P. Neumeister P. Bode K. Heissler J. Schönfelder S. (2010). EmoPics: subjektive und psychophysiologische Evaluation neuen Bildmaterials für die klinisch-biopsychologische Forschung. Zeitschrift Klin. Psychol. Psychother. 39:77.

[B81] Whitfield-Gabrieli S. Nieto-Castanon A. (2012). Conn: a functional connectivity toolbox for correlated and anticorrelated brain networks. Brain Connect. 2, 125–141. 10.1089/brain.2012.0073 22642651

[B82] Winecoff A. Labar K. S. Madden D. J. Cabeza R. Huettel S. A. (2011). Cognitive and neural contributors to emotion regulation in aging. Soc. Cogn. Affect. Neurosci. 6, 165–176. 10.1093/scan/nsq030 20385663 PMC3073384

[B83] Zaehringer J. Jennen-Steinmetz C. Schmahl C. Ende G. Paret C. (2020). Psychophysiological effects of downregulating negative emotions: insights from a meta-analysis of healthy adults. Front. Psychol. 11:470. 10.3389/fpsyg.2020.00470 32372993 PMC7177019

[B84] Zhang X. Zhu X. Wang X. Zhu X. Zhong M. Yi J.. (2014). First-episode medication-naive major depressive disorder is associated with altered resting brain function in the affective network. PLoS One 9:e85241. 10.1371/journal.pone.0085241 24416367 PMC3887023

[B85] Zwaan R. A. Etz A. Lucas R. E. Donnellan M. B. (2018). Making replication mainstream. Behav. Brain Sci. 41:e120. 10.1017/S0140525X17001972 29065933

