# Supplementary material for: Resting State Cortico-Limbic Functional Connectivity and Dispositional Use of Emotion Regulation Strategies: A Replication and Extension Study
Source: Front Behav Neurosci. 2020 Jul 28;14:128. doi: 10.3389/fnbeh.2020.00128 (PMC7399345; doi:10.3389/fnbeh.2020.00128)
Supplement: Supplementary file 1 [file Data_Sheet_1.pdf]

Supplementary Material

Resting state cortico-limbic functional connectivity and dispositional use of emotion regulation strategies: A replication and extension study

CONTENT

Supplementary Methods 4

Emotion regulation (ER) task 4

Supplementary Figure S1. Flow chart of experimental procedure 6

Supplementary Results 7

Supplementary Table S1. Results of Shapiro-Wilk test for normal distribution of all predictors 7

Supplementary Table S2. Comparison of all predictor variables separately for single experiments 8

Experiment 1 9

Supplementary Figure S2. Basic whole-brain seed-to-voxel connectivity maps during resting state for experiment 1, not restricted to PFC mask, without covariate and regressors. 9

Supplementary Table S3. Significant clusters associated with the four amygdala nuclei as seeds for experiment 1, not restricted to PFC mask (whole brain analyses), without covariate and regressors....9

Supplementary Table S4. Significant clusters associated with the four amygdala nuclei as seeds for experiment 1, restricted to PFC mask for reappraisal and suppression (aim 1)....................................10

Supplementary Table S5. Significant clusters associated with experiential reappraisal success with respective amygdala seeds for experiment 1, restricted to PFC mask (aim 2) 11

Supplementary Table S6. Significant clusters associated with neuronal reappraisal success with respective amygdala seeds for experiment 1, restricted to PFC mask (aim 3) 12

Experiment 2 13

Supplementary Figure S3. Basic whole-brain seed-to-voxel connectivity maps during resting state for experiment 2, not restricted to PFC mask, without covariate and regressors. 13

Supplementary Table S7. Significant clusters associated with the four amygdala nuclei as seeds for experiment 2, not restricted to PFC mask (whole brain analyses), without covariate and regressors..13

Supplementary Table S8. Significant clusters associated with the four amygdala nuclei as seeds for experiment 2, restricted to PFC mask for reappraisal and suppression (aim 1)......................................15

Supplementary Table S9. Significant clusters associated with experiential reappraisal success with respective amygdala seeds for experiment 2, restricted to PFC mask (aim 2) 16

Supplementary Table S10. Significant clusters associated with neuronal reappraisal success for amygdala as seeds for experiment 2, restricted to PFC mask (aim 3) 17

Experiment 3..18

Supplementary Figure S4. Basic whole-brain seed-to-voxel connectivity maps during resting state for experiment 3, not restricted to PFC mask, without covariate and regressors 18

Supplementary Table S11. Significant clusters associated with the four amygdala nuclei as seeds for experiment 3, not restricted to PFC mask (whole brain analyses), without covariate and regressors..18

Supplementary Table S12. Significant clusters associated with the four amygdala nuclei as seeds for experiment 3, restricted to PFC mask for reappraisal and suppression (aim 1)......................................20

Supplementary Table S13. Significant clusters associated with experiential reappraisal success with respective amygdala seeds for experiment 3, restricted to PFC mask (aim 2) 21

Supplementary Table S14. Significant clusters associated with neuronal reappraisal success for amygdala as seeds for experiment 3, restricted to PFC mask (aim 3) 21

Whole sample without covariate..23

Supplementary Figure S5. Basic whole-brain seed-to-voxel connectivity maps during resting state for the whole sample, not restricted to PFC mask, without covariate and regressors..................................23

Supplementary Table S15. Significant clusters associated with the four amygdala nuclei as seeds for the whole sample, not restricted to PFC mask (whole brain analyses), without covariate and regressors 23

Supplementary Table S16. Significant clusters associated with the four amygdala nuclei as seeds for the whole sample, restricted to PFC mask, without covariate, for reappraisal and suppression (aim 1) 25

Supplementary Table S17. Significant clusters associated with experiential reappraisal success with respective amygdala seeds for the whole sample, restricted to PFC mask, without covariate (aim 2) 26

Supplementary Table S18. Significant clusters associated with neuronal reappraisal success for amygdala as seeds for the whole sample, restricted to PFC mask, without covariate (aim 3) 27

Whole sample with covariate (cf. manuscript) 28

Supplementary Table S19. Significant clusters associated with the four amygdala nuclei as seeds for the whole sample, with experiment as covariate, restricted to PFC mask........28

Supplementary Table S20. Significant clusters associated with the four amygdala nuclei as seeds for the whole sample, with experiment as covariate, restricted to PFC mask for reappraisal and suppression (aim 1)........29

Supplementary Table S21. Significant clusters associated with experiential reappraisal success with respective amygdala seeds for the whole sample, with experiment as covariate, restricted to PFC mask (aim 2)........30

Supplementary Table S22. Significant clusters associated with neuronal reappraisal success for amygdala nuclei as seeds for the whole sample, with experiment as covariate, restricted to PFC mask (aim 3)........30

Differences in methods 31

Supplementary Table S23. Differences in methods between (Picó-Pérez et al., 2018) and the replication (for details see also https://osf.io/8wsgu)........31

Supplementary Table S24. Differences in methods between (Uchida et al., 2015) and the replication (for details see also https://osf.io/8wsgu)........34

Supplementary Methods

Emotion regulation (ER) task

Experiment 1:

The whole experiment took place on two days (several days apart) with one session each (Diers, Weber, Brocke, Strobel, & Schonfeld, 2014). During the first session (55 min), participants performed an adjustment measure (5 min), two runs of the ER task (30 min), an anatomical scan (7 min) and a re-exposure task (10 min). During the second session (60 min), participants performed an adjustment measure (5 min), another two runs of the ER task (20 min), the resting state measurement (10 min), a re-exposure task (10 min), and completed questionnaires.

Regarding the ER task, participants were asked to either passively view a set of negative and neutral pictures, permit or down-regulate their emotions arising in response to the pictures. During the "view" condition, participants were asked to simply view the pictures. During the "permit" condition, participants should take a close look at the pictures and permit any emotions that might arise. They were told to imagine immediately witnessing the depicted situation. However, they should not voluntarily intensify their emotions, re-interpret the situation, or distract themselves. During the "detach" condition, they were asked to "take the position of a non-involved observer, thinking about the picture in a neutral way." To achieve the detachment, participants were told to reduce personal involvement with the depicted situation, for example, by assuming personal or physical distance. Once more, participants were told not to re-interpret the situation as not real, attaching a different meaning to the situation, or distracting themselves. All participants received written instructions including examples and completed a training session outside the MR scanner which took about 15 min and consisted of 16 trials. Following this, participants were interviewed about the application of the emotion regulation strategies.

Each experimental trial consisted of a stimulation period, a rating period, and a relaxation period. In the stimulation period, a picture was presented for 8 s. Within the initial 2 s of this period, a semi-transparent overlay containing the instruction was presented in the center of the picture. Subsequently, participants rated their momentary subjective arousal (ranging from "not at all aroused" to "very highly aroused"; 3 s). Following this, a fixation cross was presented for 12 s (relaxation period) with another arousal rating at the end of this period (3 s). The total duration of a single trial was 30 s on average. Each of the four runs of the ER task consisted of 30 trials: 10 trials each for "permit negative," "view negative," and "view neutral" (first session), and "detach negative", "view negative", and "view neutral" (second session), respectively.

Experiment 2:

The whole experiment took place on two days (one week apart) with one session each (Diers et al., in preparation; Gärtner et al., 2019; Scheffel et al., 2019). During the first session (60 min), participants performed an adjustment measure (5 min), four runs of the ER task (36 min), an anatomical scan (8 min) and a re-exposure task (10 min). During the second session (25–35 min), participants performed an adjustment measure (5 min), the resting state measurement (8 min), a re-exposure task (10 min), and completed questionnaires.

Regarding the ER task, participants were asked to either permit or down-regulate their emotions arising in response to a set of negative and neutral pictures. Instructions for "permit" and "detach" strategies corresponded to the instructions in experiment 1 (see above). All participants received written instructions including examples and completed a training session outside the MR scanner which took about 15 min and consisted of 16 trials. Following this, participants were interviewed about the application of the ER strategies.

Each experimental trial consisted of a stimulation period and a relaxation period. In the stimulation period, a picture was presented for 10 s. Within the initial 2 s of this period, a semi-transparent overlay containing the instruction was presented in the center of the picture. Subsequently, participants should relax while viewing a fixation cross for 16–24 s (average: 20 s, Relaxation period). The total duration of a single trial was 30 s on average. Each of the four runs of the ER task consisted of 16 trials with four trials of each condition (permit neutral, permit negative, detach neutral, detach negative), after which the participants performed retrospective arousal ratings (ranging from "not at all aroused" to "very highly aroused").

Experiment 3:

The experiment took place on two days (one week apart) with one session each (Diers et al., in preparation; Gärtner et al., 2019; Scheffel et al., 2019). During the first session (70 min), an adjustment measure (5 min), four runs of the ER task (44 min), an anatomical scan (8 min) and a re-exposure task (12 min). During the second session (30–40 min), participants performed an adjustment measure (5 min), the resting state measurement (8 min), a re-exposure task (12 min), and completed questionnaires.

Regarding the ER task, participants were asked to permit, down-regulate, or intensify their emotions arising in response to a set of negative and neutral pictures. Instructions for "permit" and "detach" strategies corresponded to the instructions in experiment 1 (see above). During the "intensify" condition, participants were instructed to intensify their upcoming emotions by amplifying physical changes and imagining to participate in the depicted situation. All participants received written instructions including examples and completed a training session outside the MR scanner which took about 15 min and consisted of 24 trials. Following this, participants were interviewed about the application of the ER strategies.

Each experimental trial consisted of a stimulation period and a relaxation period. In the stimulation period, a picture was presented for 8 s. Within the initial 2 s of this period, a semi-transparent overlay containing the instruction was presented in the center of the picture. Subsequently, participants should relax while viewing a fixation cross for 12–20 s (average: 16 s, Relaxation period). The total duration of one trial was 24 s on average. Each of the four runs of the ER task consisted of 24 trials with four trials of each condition (permit neutral, permit negative, detach neutral, detach negative, intensify negative, intensify neutral), after which the participants performed retrospective arousal ratings (ranging from "not aroused" to "very highly aroused").

For more information on stimuli and design specification, please refer to the preregistration of this study (https://osf.io/xmz6j/).

Supplementary Figure S1. Flow chart of experimental procedure

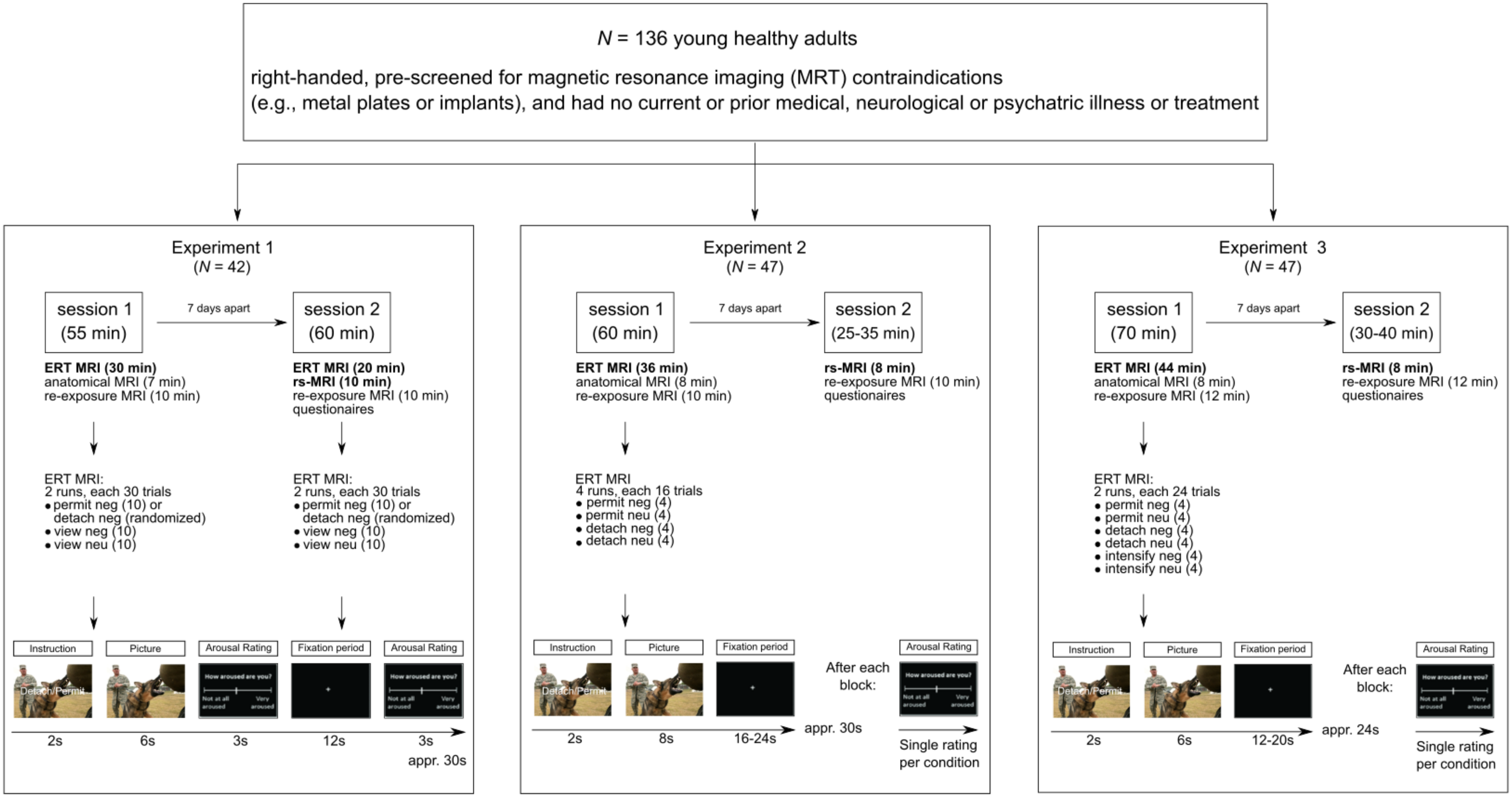

Supplementary Results

Supplementary Table S1. Results of Shapiro-Wilk test for normal distribution of all predictors

Variable	*M*	*SD*	Test statistic *W*	*p*-value
ERQ Suppression	3.4	1.2	0.976	.065
ERQ Reappraisal	4.8	0.8	0.979	.117
PANAS PE	41.8	5.8	0.988	.492
PANAS NE *	22	5.8	0.959	.003
Rating negative permit *	13.6	59.8	0.906	< .001
Rating negative detach *	-15.4	68.9	0.955	.002

Note. * Variable is not normally distributed.

Supplementary Table S2. Comparison of all predictor variables separately for single experiments

Variable ($M \pm SD$)	*Experiment 1*	*Experiment 2*	*Experiment 3*	ANOVA between experiments *
Age	24.5 ± 5.6	24 ± 3.3	24.7 ± 4.1	$F(2,103) = 0.22$, $p = .80$
Gender (male/female)	9 / 17	14 / 26	19 / 21	$F(2,103) = 0.82$, $p = .44$
ERQ Suppression	3.0 ± 1.2	3.5 ± 1.2	3.6 ± 1.2	$F(2,99) = 1.73$, $p = .18$
ERQ Reappraisal	4.6 ± 0.7	4.8 ± 0.9	4.9 ± 0.8	$F(2,98) = 0.97$, $p = .38$
PANAS PE	43.5 ± 6.4	41.8 ± 5.9	40.6 ± 5.2	$F(2,100) = 2.08$, $p = .13$
PANAS NE	22.2 ± 5.3	22.6 ± 6.2	21.2 ± 5.8	$H(2) = 0.64$, $p = .72$
Experiential arousal rating				
Rating negative permit	5.8 ± 43.5	20.5 ± 71	12.7 ± 58.9	$H(2) = 4.68$, $p = .10$
Rating negative detach	-57.2 ± 60.9	1.4 ± 74.1	-2.1 ± 58.9	$H(2) = 14.76$, $p < .001$
Experiential reappraisal success (Δ neg_{permit} - neg_{detach})	67.5 ± 52.6	19.1 ± 34.1	14.8 ± 36	$H(2) = 23.79$, $p < .001$
Neuronal reappraisal success (Δ neg_{permit} - $neg_{detach)}$				
Activity AMY BLA left	1.3 ± 2.0	1.8 ± 5.2	4.2 ± 9.3	$H(2) = 1.07$, $p = .59$
Activity AMY BLA right	1.3 ± 2.5	1.9 ± 5.6	3.7 ± 8.2	$H(2) = 1.75$, $p = .42$
Activity AMY CMA left	2.0 ± 3	3.7 ± 8	3.9 ± 9.5	$H(2) = 1.33$, $p = .51$
Activity AMY CMA right	2 ± 2.9	2.6 ± 8.3	3.8 ± 9.9	$H(2) = 0.46$, $p = .79$

Note. AMY, Amygdala; BLA, basolateral; CM, centromedial; * A Kruskal-Wallis rank sum test was performed, if variable is not normal distributed

Experiment 1

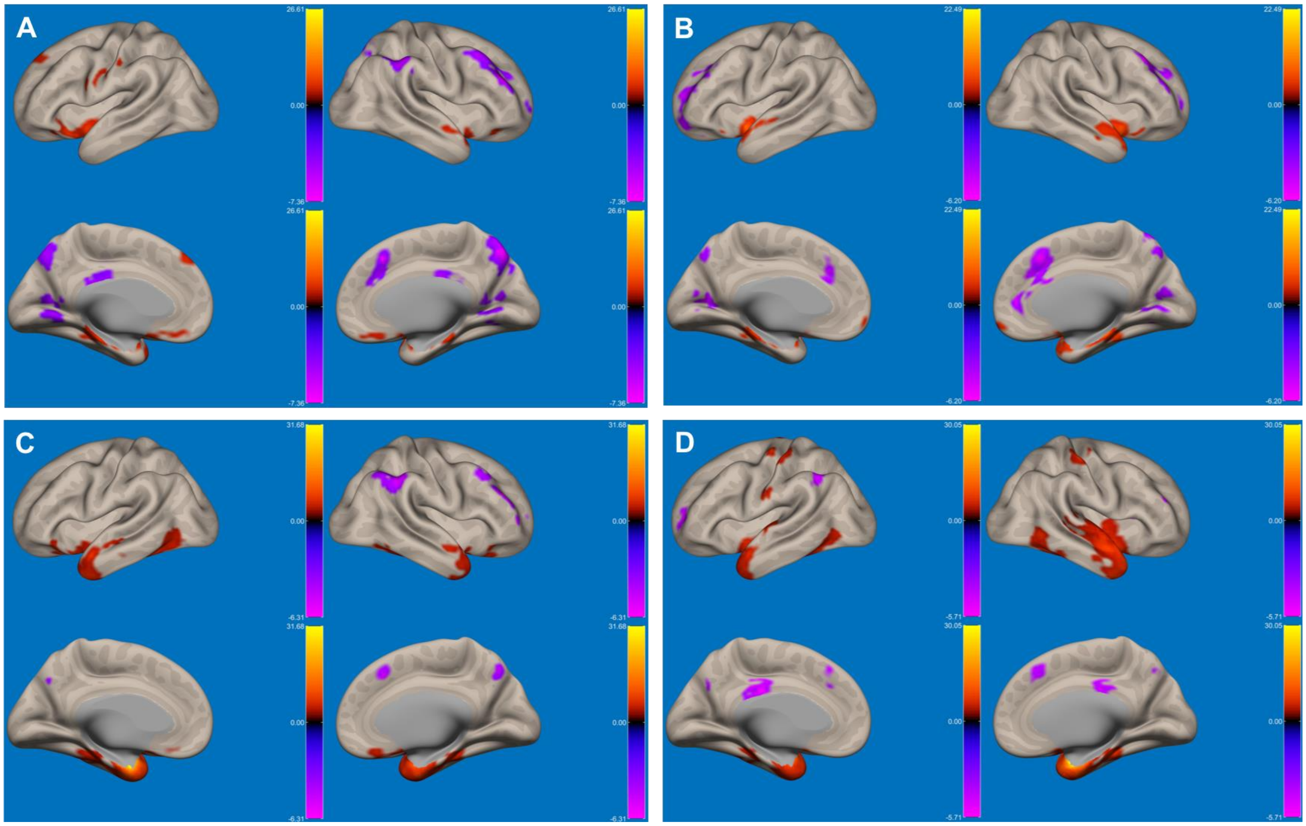

Supplementary Figure S2. Basic whole-brain seed-to-voxel connectivity maps during resting state for experiment 1, not restricted to PFC mask, without covariate and regressors. Blobs depict regions positively (red) or negatively (blue) coupled with left centromedial amygdala (A), right centromedial amygdala (B), left basolateral amygdala (C), and right basolateral amygdala (D). Results are presented on a voxel-level of $p < 0.001$ uncorrected, FWE cluster-level corrected for multiple comparisons ($p < 0.05$). Connectivity maps are presented on a rendered brain surface from CONN.

Supplementary Table S3. Significant clusters associated with the four amygdala nuclei as seeds for experiment 1, not restricted to PFC mask (whole brain analyses), without covariate and regressors

Region	H	x	y	z	*k*	*T*	*p*-FWE
Left centromedial amygdala							
Amygdala/Cerebellum	L	-18	-4	-18	1202	45.34	<.001
Amygdala/Parahippocampal Gyrus	R	22	-2	-16	672	13.78	<.001
Precentral Gyrus	L	-54	2	20	1	6.45	<.001
Inferior Orbitofrontal Gyrus	L	-32	20	-16	1	6.37	<.001
Parahippocampal Gyrus	L	-28	-34	-14	1	6.37	<.001
Insula	L	-30	16	-18	1	6.36	<.001
Right centromedial amygdala							

Amygdala/Parahippocampal Gyrus/Superior Temporal Pole	R	20	-4	-16	1443	48.29	<.001
Hippocampus/Insula	L	-20	-8	-18	577	48.29	<.001
Cerebellum 3	L	-12	-28	-22	60	7.76	<.001
Olfactory Gyrus	R	4	4	-14	10	7.62	<.001
Left basolateral amygdala							
Amygdala/Middle Temporal Pole/Superior Temporal Pole	L	-22	-4	-26	1984	38.19	<.001
Hippocampus/Parahippocampal Gyrus	R	22	-8	-20	977	12.98	<.001
Fusiform Gyrus	L	-42	-48	-20	36	8.54	<.001
Inferior Orbitofrontal Gyrus	L	-28	28	-20	8	7.13	<.001
Superior Temporal Pole	R	38	12	-28	34	7.07	<.001
Parahippocampal Gyrus	R	34	-24	-22	21	6.87	<.001
Fusiform Gyrus	L	-38	-42	-18	4	6.83	<.001
Fusiform Gyrus	R	40	-38	-16	2	6.77	<.001
Middle Temporal Pole	R	38	18	-44	5	6.73	<.001
Olfactory Gyrus	R	6	10	-12	1	6.55	<.001
Middle Temporal Gyrus	R	50	-4	-16	2	6.44	<.001
Right basolateral amygdala							
Amygdala/Superior Temporal Pole/ Middle Temporal Pole	R	24	-2	-22	2832	35.42	<.001
Hippocampus/Middle Temporal Pole/Middle Temporal Gyrus	L	-22	-6	-20	1029	16.03	<.001
Precentral Gyrus	L	-42	-16	68	39	9.81	<.001
Rectus	L	-4	12	-20	3	7.43	<.001
Inferior Temporal Gyrus	R	40	-52	-14	19	7.24	<.001
Postcentral Gyrus	R	46	-24	60	13	6.89	<.001
Inferior Temporal Gyrus	R	52	-56	-6	1	6.45	<.001
Superior Temporal Gyrus	R	60	-16	2	1	6.43	<.001
Insula	R	36	-20	6	1	6.41	<.001

Note. Significance threshold for seed-to-voxel analyses set at $p < .05$ FWE-corrected. Coordinates are given in MNI space. Amy, amygdala; R, right; L, left; H, Hemisphere.

Supplementary Table S4. Significant clusters associated with the four amygdala nuclei as seeds for experiment 1, restricted to PFC mask for reappraisal and suppression (aim 1)

Region	H	x	y	z	*k*	*T*	*p*-uncorr
Reappraisal							
Left centromedial amygdala							
Insula	R	32	6	18	6	3.96	<.001
Rolandic Operculum	R	52	6	16	2	3.59	.001
Right centromedial amygdala							
Insula	L	-38	-16	22	3	3.87	<.001
Orbitofrontal Medial Gyrus	L	-6	56	-8	4	3.79	<.001
Precentral Gyrus	R	50	-2	26	4	3.79	<.001

Rolandic Operculum	R	44	-18	22	8	3.70	.001
Precuneus	R	4	-52	26	1	3.67	.001
Insula	R	36	-6	18	4	3.65	.001
Insula	L	-34	-14	16	1	3.50	.001
Left basolateral amygdala							
Insula	R	34	-4	18	49	5.01	<.001
Supplemental Motor Area	R	8	-22	58	29	4.97	<.001
Putamen	R	38	-4	0	8	4.36	<.001
Orbitofrontal Medial Gyrus	L	-10	58	-6	6	4.06	<.001
Middle Frontal Gyrus	R	40	8	58	16	3.78	<.001
Superior Frontal Gyrus	R	20	-16	62	3	3.72	.001
Right basolateral amygdala							
Insula	R	36	-10	22	22	4.27	<.001
Orbitofrontal Middle Gyrus	L	-24	44	-2	2	4.14	<.001
Supplemental Motor Area	R	6	-22	66	25	4.06	<.001
Paracentral Lobule	L	-2	-26	66	3	3.64	.001
Inferior Frontal Gyrus Triangularis	L	-60	34	4	1	3.58	.001
Suppression							
Left centromedial amygdala							
Superior Frontal Gyrus	L	-16	10	48	26	5.40	<.001
Right centromedial amygdala							
No suprathreshold clusters							
Left basolateral amygdala							
Middle Frontal Gyrus	L	-34	16	60	8	3.94	<.001
Middle Frontal Gyrus	L	-32	22	54	4	3.65	.001
Inferior Frontal Gyrus Triangularis	L	-54	40	28	1	3.54	.001
Inferior Frontal Gyrus Triangularis	L	-58	36	26	1	3.50	.001
Right basolateral amygdala							
Insula	L	-30	-8	18	4	3.65	.001
Precuneus	R	18	-42	40	1	3.45	.001

Note. Significance threshold for seed-to-voxel analyses set at $p < .001$ uncorrected. Coordinates are given in MNI space. Amy, amygdala; R, right; L, left; H, Hemisphere.

Supplementary Table S5. Significant clusters associated with experiential reappraisal success with respective amygdala seeds for experiment 1, restricted to PFC mask (aim 2)

Region	H	x	y	z	*k*	*T/F*	*p*-uncorr
Left amygdala (BLA + CMA)							
Superior Frontal Gyrus	R	24	28	48	65	4.84	<.001
Middle Cingulum	L	-12	26	30	15	4.58	<.001
Middle Frontal Gyrus	R	44	20	42	2	3.79	<.001
Superior Frontal Gyrus	R	12	42	50	5	3.69	.001
Middle Cingulum	R	20	-10	42	3	3.67	.001
Superior Medial Frontal Gyrus	R	12	40	46	3	3.67	.001

Superior Medial Frontal Gyrus	R	8	32	62	2	3.62	.001
Inferior Frontal Gyrus Triangularis	R	36	24	14	2	3.60	.001
Precuneus	L	2	-60	26	1	3.49	.001
Right amygdala (BLA + CMA)							
Anterior Cingulum	L	-12	26	30	15	5.42	<.001
Superior Medial Frontal Gyrus	R	10	40	42	14	4.20	<.001
Amygdala (Any nucleus)							
Superior Frontal Gyrus	L	-20	-2	68	27	6.10	<.001
Superior Medial Frontal Gyrus	R	12	40	44	8	5.81	<.001
Supplemental Motor Area	L	-8	-8	58	9	5.79	<.001
Middle Frontal Gyrus	R	26	28	46	3	5.65	<.001

Note. Significance threshold for seed-to-voxel analyses set at $p < .001$ uncorrected. Coordinates are given in MNI space. Amy, amygdala; R, right; L, left; H, Hemisphere.

Supplementary Table S6. Significant clusters associated with neuronal reappraisal success with respective amygdala seeds for experiment 1, restricted to PFC mask (aim 3)

Region	H	x	y	*z*	*k*	*F*	*p*-uncorr
Amygdala (Any nucleus)							
Middle Frontal Gyrus	L	-40	28	46	68	7.40	<.001
Caudate	R	18	-2	28	5	7.25	<.001
Paracentral Lobule	L	-8	-14	80	41	6.74	<.001
Inferior Orbitofrontal Gyrus	L	-30	26	-22	3	6.26	<.001
Supplementary Motor Area	L	-2	-4	66	5	6.23	<.001
Precentral Gyrus	R	66	6	16	3	6.18	<.001
Heschl Gyrus	L	-46	-14	10	5	6.14	<.001
Supplementary Motor Area	R	2	-14	52	19	6.09	<.001
Paracentral Lobule	L	-2	-24	74	8	6.04	<.001
Superior Frontal Gyrus	R	22	48	42	12	6.01	<.001
Supplementary Motor Area	R	2	-6	66	6	5.75	<.001
Supplementary Motor Area	R	12	2	52	2	5.60	.001
Middle Frontal Gyrus	R	34	36	44	2	5.52	.001
Insula	L	-28	22	-12	1	5.48	.001
Inferior Orbitofrontal Gyrus	R	36	40	-4	1	5.34	.001
Precentral Gyrus	R	52	2	22	1	5.31	.001

Note. Significance threshold for seed-to-voxel analyses set at $p < .001$ uncorrected. Coordinates are given in MNI space. Amy, amygdala; R, right; L, left; H, Hemisphere.

Experiment 2

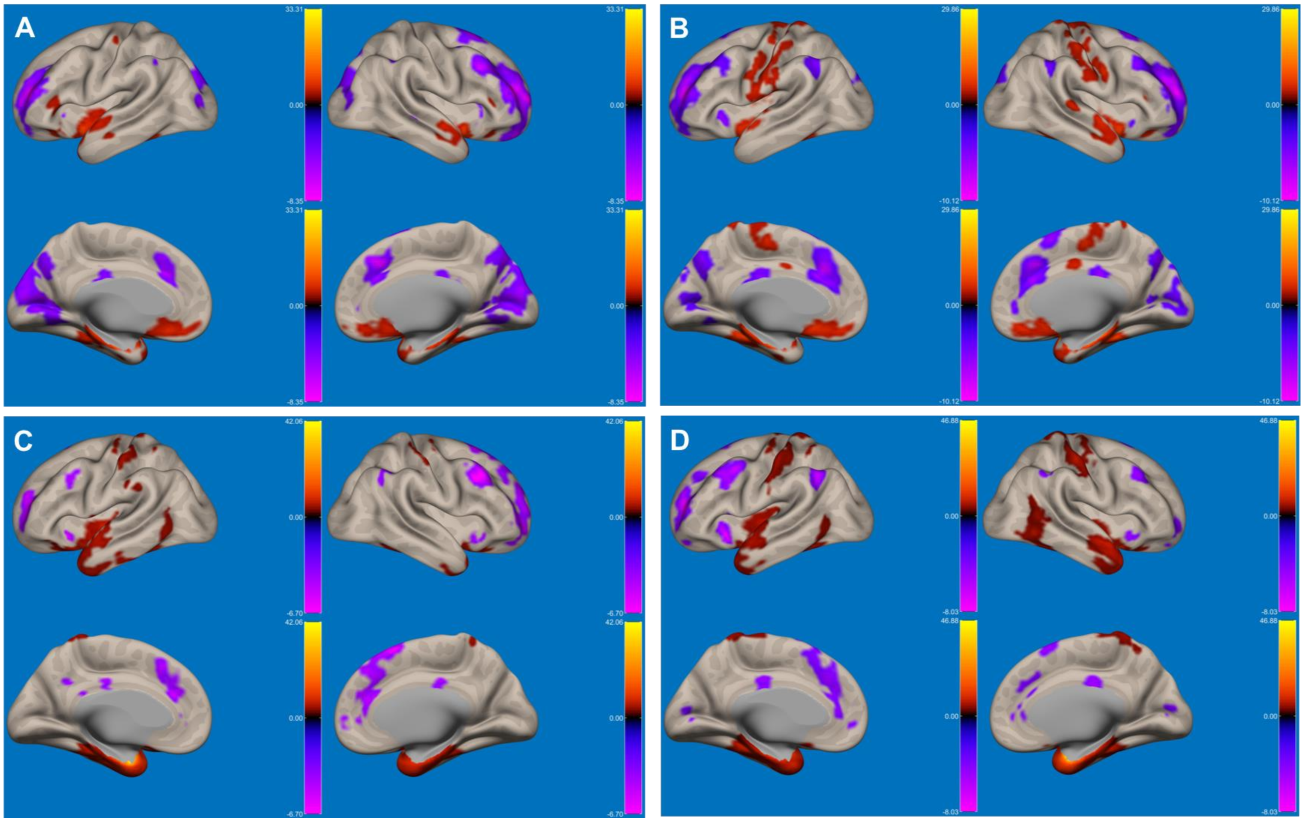

Supplementary Figure S3. Basic whole-brain seed-to-voxel connectivity maps during resting state for experiment 2, not restricted to PFC mask, without covariate and regressors. Blobs depict regions positively (red) or negatively (blue) coupled with left centromedial amygdala (A), right centromedial amygdala (B), left basolateral amygdala (C), and right basolateral amygdala (D). Results are presented on a voxel-level of $p < 0.001$ uncorrected, FWE cluster-level corrected for multiple comparisons ($p < 0.05$). Connectivity maps are presented on a rendered brain surface from CONN.

Supplementary Table S7. Significant clusters associated with the four amygdala nuclei as seeds for experiment 2, not restricted to PFC mask (whole brain analyses), without covariate and regressors

Region	H	x	y	z	*k*	*T*	*p*-FWE
Left centromedial amygdala							
Amygdala/Hippocampus	R	-18	-4	-18	5028	63.21	<.001
Fusiform Gyrus	L	-38	-44	-18	52	7.75	<.001
Inferior Frontal Gyrus Triangularis	R	58	36	8	25	6.94	<.001
Orbitomedial Frontal Gyrus	L	-8	30	-14	22	6.76	<.001
Cerebellum 3	R	10	-40	-24	33	6.68	<.001
Lingual Gyrus	L	-14	-30	-4	13	6.62	<.001
Thalamus	R	14	-30	-2	15	6.62	<.001
Olfactory Gyrus	R	2	22	-14	16	6.24	<.001

Orbitomedial Frontal Gyrus	L	-2	42	-14	19	6.14	<.001
Superior Temporal Gyrus	R	56	-8	-12	8	6.11	<.001
Inferior Orbitofrontal Gyrus	L	-26	30	-14	1	5.87	<.001
Middle Temporal Gyrus	L	-62	-10	-10	1	5.79	<.001
Middle Temporal Pole	R	58	6	-20	1	5.79	<.001
Right centromedial amygdala							
Parahippocampal Gyrus/ Cerebellum 3/ Fusiform Gyrus	R	18	-4	-18	2729	53.89	<.001
Amygdala/Parahippocampal Gyrus/ Fusiform Gyrus	L	-18	-6	-18	2251	20.13	<.001
Olfactory Gyrus/Medial Orbitofrontal Gyrus	L	-2	22	-14	67	7.62	<.001
Inferior Frontal Gyrus Triangularis	R	56	40	10	40	7.58	<.001
Medial Orbitofrontal Gyrus	R	2	42	-12	42	7.01	<.001
Superior Temporal Pole	R	44	20	-22	41	6.63	<.001
Supplementary Motor Area	L/R	-2	-20	58	25	6.39	<.001
Postcentral Gyrus	R	50	-14	38	12	6.33	<.001
Inferior Orbitofrontal Gyrus	L	-26	28	-14	3	6.31	<.001
Postcentral Gyrus	L	-54	-12	38	16	6.17	<.001
Lingual Gyrus	R	14	-32	-2	5	6.09	<.001
Postcentral Gyrus	R	54	-4	32	6	6.06	<.001
Precentral Gyrus	L	-32	-28	64	2	6.02	<.001
Middle Temporal Pole	R	26	10	-40	4	5.98	<.001
Superior Orbitofrontal Gyrus	R	20	28	-14	6	5.97	<.001
Middle Temporal Pole	R	40	20	-34	1	5.8	<.001
Left basolateral amygdala							
Amygdala/Hippocampus/Inferior Temporal Gyrus	L	-24	-2	-26	3861	49.01	<.001
Amygdala/Parahippocampal Gyrus	R	26	-2	-16	2309	12.53	<.001
Precentral Gyrus	R	28	-12	72	22	6.88	<.001
Insula/Superior Temporal Gyrus	L	-38	-12	-2	35	6.61	<.001
Fusiform Gyrus	R	44	-44	-16	13	6.41	<.001
Thalamus	R	14	-26	0	2	6.09	<.001
Postcentral Gyrus	L	-54	-28	56	17	6.03	<.001
Cerebellum 3	R	14	-22	-28	2	5.96	<.001
Cerebellum 3	R	10	-34	-26	4	5.92	<.001
Pallidum	L	-8	2	-10	1	5.88	<.001
Parahippocampal Gyrus	R	6	-14	-24	2	5.82	<.001
Paracentral Lobule	L	-10	-38	78	1	5.81	<.001
Putamen	L	-34	0	-4	1	5.79	<.001
Right basolateral amygdala							
Parahippocampal Gyrus/Middle Temporal Pole/Middle Temporal Gyrus	R	24	-2	-26	3984	54.81	<.001
Hippocampus/Amygdala/Hippocampus	L	-22	-8	-20	3045	15.03	<.001
Fusiform Gyrus	R	44	-44	-16	100	7.69	<.001
Postcentral Gyrus	R	48	-24	52	53	7.36	<.001
Superior Temporal Gyrus	L	-44	-14	-2	37	7.15	<.001
Paracentral Lobule	L	-12	-30	80	30	7.06	<.001
Precentral Gyrus	R	48	-14	58	49	7.05	<.001

Cerebellum 3	R	8	-22	-22	14	6.58	<.001
Inferior Temporal Gyrus	R	56	-60	-6	18	6.57	<.001
Cerebellum 3	R	10	-24	-36	4	6.07	<.001
Olfactory Gyrus	R	6	4	-18	4	6.07	<.001
Precentral Gyrus	R	16	-28	76	1	5.8	<.001
Postcentral Gyrus	L	-54	-24	56	1	5.76	<.001

Note. Significance threshold for seed-to-voxel analyses set at $p < .05$ FWE-corrected. Coordinates are given in MNI space. Amy, amygdala; R, right; L, left; H, Hemisphere.

Supplementary Table S8. Significant clusters associated with the four amygdala nuclei as seeds for experiment 2, restricted to PFC mask for reappraisal and suppression (aim 1)

Region	H	x	y	z	*k*	*T*	*p*-uncorr
Reappraisal							
Left centromedial amygdala							
Superior Frontal Gyrus	R	22	32	36	17	4.28	<.001
Middle Frontal Gyrus	R	36	40	14	2	3.47	.001
Right centromedial amygdala							
Middle Frontal Gyrus	R	36	46	16	16	3.84	<.001
Middle Frontal Gyrus	R	32	30	44	14	3.68	<.001
Superior Frontal Gyrus	R	22	40	38	1	3.42	.001
Supplemental Motor Area	L	-14	-2	46	2	3.35	.001
Orbitofrontal Medial Gyrus	R	14	50	-10	2	3.33	.001
Left basolateral amygdala							
Middle Frontal Gyrus	L	-30	44	14	31	4.98	<.001
Middle Frontal Gyrus	R	36	12	50	14	4.10	<.001
Superior Frontal Gyrus/Middle Frontal Gyrus	R	22	34	34	36	3.87	<.001
Middle Frontal Gyrus	R	28	18	46	16	3.78	<.001
Superior Frontal Gyrus	R	22	52	10	7	3.75	<.001
Superior Frontal Gyrus	R	22	48	-2	12	3.71	<.001
Middle Frontal Gyrus	R	56	30	36	3	3.51	.001
Inferior Frontal Gyrus Triangularis	R	44	20	10	1	3.50	.001
Precentral Gyrus	L	-32	4	42	4	3.49	.001
Superior Frontal Gyrus	R	28	64	6	1	3.45	.001
Middle Frontal Gyrus	R	38	34	22	1	3.39	.001
Right basolateral amygdala							
Middle Frontal Gyrus	L	-28	54	16	33	4.35	<.001
Superior Frontal Gyrus	R	18	10	58	29	4.08	<.001
Middle Frontal Gyrus	L	-32	4	62	12	3.82	<.001
Middle Frontal Gyrus	L	-42	40	16	22	3.66	<.001
Anterior Cingulum	L	-2	42	18	1	3.48	.001
Middle Cingulum	R	10	32	30	2	3.34	.001
Insula	L	-36	22	-4	1	3.34	.001
Suppression							
Left centromedial amygdala							
Precuneus	L	-12	-44	42	6	4.07	<.001

Superior Frontal Gyrus	L	-16	-2	74	2	3.41	.001
Right centromedial amygdala							
Rectus	R	10	48	-18	19	4.55	<.001
Orbitofrontal Superior Gyrus	R	18	32	-28	8	4.16	<.001
Middle Cingulum	R	10	4	32	5	3.85	<.001
Insula	L	-36	14	-16	4	3.56	.001
Posterior Cingulum	L	-4	-38	30	1	3.39	.001
Inferior Frontal Gyrus Opercularis	L	-38	20	34	1	3.37	.001
Left basolateral amygdala							
Orbitofrontal Inferior Gyrus	R	38	18	-20	26	4.66	<.001
Supplemental Motor Area	R	6	2	68	48	4.03	<.001
Precentral Gyrus	R	40	-2	-36	6	3.70	<.001
Middle Frontal Gyrus	L	-34	38	16	2	3.58	<.001
Supplemental Motor Are	L	-14	-16	56	1	3.36	.001
Right basolateral amygdala							
Supplemental Motor Area	L	-6	-10	62	18	4.04	<.001
Insula	L	-40	-2	4	22	3.97	<.001
Supplemental Motor Area	R	14	-6	50	6	3.51	.001

Note. Significance threshold for seed-to-voxel analyses set at $p < .001$ uncorrected. Coordinates are given in MNI space. Amy, amygdala; R, right; L, left; H, Hemisphere.

Supplementary Table S9. Significant clusters associated with experiential reappraisal success with respective amygdala seeds for experiment 2, restricted to PFC mask (aim 2)

Region	H	x	y	*z*	*k*	*T/F*	*p*-uncorr
Left amygdala (BLA + CMA)							
Middle Cingulum	L	-10	20	32	63	4.93	<.001
Superior Frontal Gyrus	L	-12	40	32	13	4.05	<.001
Superior Medial Frontal Gyrus	L	-10	50	4	21	3.85	<.001
Superior Frontal Gyrus	R	22	52	-4	3	3.79	<.001
Middle Frontal Gyrus	R	38	46	4	33	3.77	<.001
Superior Frontal Gyrus	R	20	34	30	5	3.54	.001
Middle Cingulum	L	-8	-8	34	5	3.49	.001
Right amygdala (BLA + CMA)							
Middle Cingulum	L	-12	22	30	-12	-12	<.001
Superior Frontal Gyrus/Superior Medial Frontal Gyrus/Anterior Cingulum	R	20	30	32	20	20	<.001
Superior Medial Frontal Gyrus	L	-2	58	30	-2	-2	<.001
Superior Medial Frontal Gyrus	L	-2	40	32	-2	-2	<.001
Superior Medial Frontal Gyrus	L	-12	48	6	-12	-12	<.001
Inferior Frontal Gyrus Triangularis	R	44	20	4	44	44	.001
Anterior Cingulum	L	-8	4	28	-8	-8	.001
Inferior Frontal Gyrus Triangularis	R	44	26	4	44	44	.001
Rectus	L	-2	26	-18	-2	-2	.001
Middle Frontal Gyrus	R	34	6	50	34	34	.001

Amygdala (Any nucleus)							
Anterior Cingulum	R	4	42	26	41	6.50	<.001
Middle Frontal Gyrus	L	-32	32	52	14	6.49	<.001
Supplementary Motor Area	R	16	-4	68	8	6.06	<.001
Precentral Gyrus	R	40	2	50	8	5.26	.001
Superior Medial Frontal Gyrus	L	-2	42	32	4	4.96	.001
Superior Temporal Pole	L	-34	12	-18	1	4.95	.001
Middle Frontal Gyrus	R	32	50	2	1	4.93	.001

Note. Significance threshold for seed-to-voxel analyses set at $p < .001$ uncorrected. Coordinates are given in MNI space. Amy, amygdala; R, right; L, left; H, Hemisphere.

Supplementary Table S10. Significant clusters associated with neuronal reappraisal success for amygdala as seeds for experiment 2, restricted to PFC mask (aim 3)

Region	H	x	y	*z*	*k*	*F*	*p*-uncorr
Amygdala (Any nucleus)							
Middle Frontal Gyrus	R	38	26	32	1	5.14	.001
Middle Cingulum	R	8	-40	40	1	5.01	.001

Note. Significance threshold for seed-to-voxel analyses set at $p < .001$ uncorrected. Coordinates are given in MNI space. Amy, amygdala; R, right; L, left; H, Hemisphere.

Experiment 3

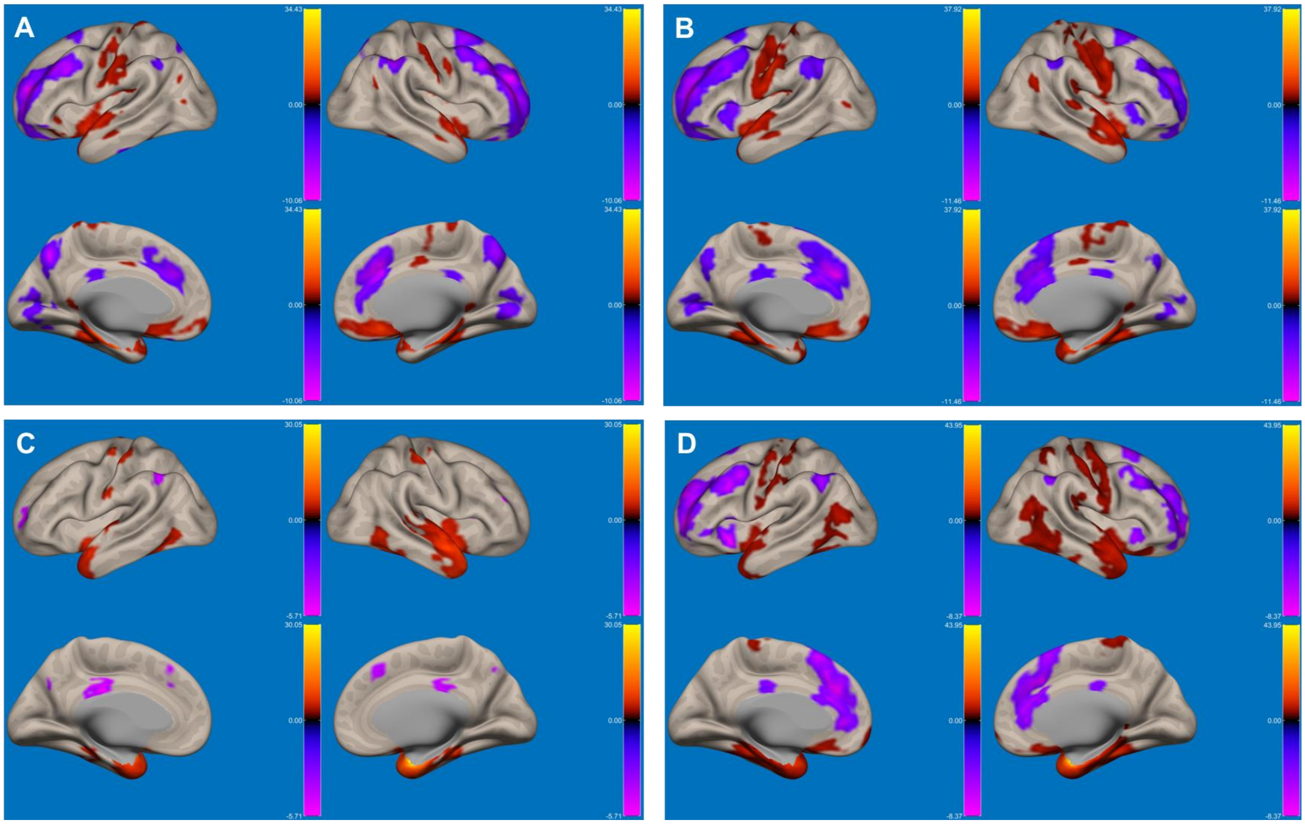

Supplementary Figure S4. Basic whole-brain seed-to-voxel connectivity maps during resting state for experiment 3, not restricted to PFC mask, without covariate and regressors. Blobs depict regions positively (red) or negatively (blue) coupled with left centromedial amygdala (A), right centromedial amygdala (B), left basolateral amygdala (C), and right basolateral amygdala (D). Results are presented on a voxel-level of $p < 0.001$ uncorrected, FWE cluster-level corrected for multiple comparisons ($p < 0.05$). Connectivity maps are presented on a rendered brain surface from CONN.

Supplementary Table S11. Significant clusters associated with the four amygdala nuclei as seeds for experiment 3, not restricted to PFC mask (whole brain analyses), without covariate and regressors

Region	H	x	y	z	*k*	*T*	*p*-few
Left centromedial amygdala							
Amygdala/Hippocampus/ Parahippocampal Gyrus	L	-18	-6	-18	5638	59.27	<.001
Superior Temporal Pole	L	-40	22	-26	139	7.77	<.001
Thalamus	R	12	-34	2	51	7.71	<.001
Fusiform Gyrus	R	40	-42	-16	14	6.73	<.001
Inferior Orbitofrontal Gyrus	R	28	30	-12	13	6.62	<.001
Middle Temporal Gyrus	R	60	-4	-16	34	6.27	<.001

Middle Temporal Gyrus	L	-60	-4	-10	19	6.09	<.001
Postcentral Gyrus	L	-58	-14	38	10	6.05	<.001
Middle Occipital Gyrus	R	56	-66	24	5	6.01	<.001
Inferior Orbitofrontal Gyrus	L	-30	28	-14	2	5.84	<.001
Postcentral Gyrus	R	58	-6	38	1	5.78	<.001
Right centromedial amygdala							
Parahippocampal Gyrus/Amygdala/Gyrus Rectus	R	18	-4	-18	6637	61.47	<.001
Postcentral Gyrus	L	-58	-14	38	365	8.48	<.001
Precentral Gyrus	R	54	-6	38	192	8.06	<.001
Medial Orbitofrontal Gyrus	R	0	60	-10	133	7.89	<.001
Middle Temporal Gyrus	L	-62	-4	-12	161	7.42	<.001
Insula	R	36	-6	16	22	6.7	<.001
Inferior Temporal Gyrus	R	50	-54	-22	14	6.55	<.001
Superior Temporal Pole	L	-48	20	-22	15	6.38	<.001
Superior Temporal Pole	L	-54	8	-14	5	6.16	<.001
Thalamus	R	12	-34	2	8	6.09	<.001
Middle Temporal Gyrus	R	52	-16	-16	1	6.02	<.001
Precentral Gyrus	R	50	-16	50	10	5.97	<.001
Insula	L	-36	-16	20	6	5.88	<.001
Precentral Gyrus	R	44	-12	50	5	5.84	<.001
Cerebellum 4 5	L	-14	-46	-18	2	5.76	<.001
Middle Temporal Pole	L	-42	18	-36	1	5.75	<.001
Left basolateral amygdala							
Amygdala/Hippocampus	L	-22	-2	-26	6934	55.57	<.001
Lingual Gyrus	R	12	-36	0	34	7.17	<.001
Hippocampus	L	-14	-36	0	17	6.45	<.001
Inferior Temporal Gyrus	L	-50	-58	-18	16	6.42	<.001
Middle Temporal Gyrus	R	44	-64	14	11	6.28	<.001
Inferior Temporal Gyrus	R	52	-50	-14	4	5.89	<.001
Paracentral Lobule	R	6	-40	78	1	5.82	<.001
Right basolateral amygdala							
Hippocampus/Middle Temporal Pole/Superior Temporal Pole	R	26	-2	-24	5278	59.8	<.001
Hippocampus/Superior Temporal Pole/Parahippocampal Gyrus	L	-22	-8	-20	3020	20.64	<.001
Superior Frontal Gyrus	R	34	-4	64	113	7.64	<.001
Precentral Gyrus	R	50	-14	56	69	7.23	<.001
Rectus	R	0	28	-16	29	6.86	<.001
Olfactory Gyrus	R	6	6	-16	31	6.78	<.001
Middle Occipital Gyrus	L	-54	-72	2	72	6.66	<.001
Inferior Temporal Gyrus	L	-50	-58	-16	22	6.51	<.001
Postcentral Gyrus	L	-58	-14	40	18	6.32	<.001
Middle Temporal Gyrus	R	44	-62	16	19	6.28	<.001
Precentral Gyrus	R	32	-28	68	19	6.23	<.001
Postcentral Gyrus	R	52	-28	56	4	6.04	<.001
Inferior Temporal Gyrus	R	52	-64	-10	12	5.97	<.001
Postcentral Gyrus	L	-46	-14	56	3	5.94	<.001
Parahippocampal Gyrus	L	-8	-18	-24	1	5.92	<.001
Cerebellum 3	R	16	-26	-28	5	5.91	<.001

Note. Significance threshold for seed-to-voxel analyses set at $p < .05$ FWE-corrected. Coordinates are given in MNI space. Amy, amygdala; R, right; L, left; H, Hemisphere.

Supplementary Table S12. Significant clusters associated with the four amygdala nuclei as seeds for experiment 3, restricted to PFC mask for reappraisal and suppression (aim 1)

Region	H	x	y	z	*k*	*T*	*p*-uncorr
Reappraisal							
Left centromedial amygdala							
Inferior Orbitofrontal Gyrus	R	38	30	-2	7	3.72	<.001
Superior Frontal Gyrus	L	-24	58	14	7	3.56	.001
Right centromedial amygdala							
Insula	R	32	18	10	3	3.76	<.001
Insula	L	-40	8	6	1	3.36	.001
Superior Frontal Gyrus	R	26	64	14	2	3.36	.001
Superior Frontal Gyrus	R	28	64	18	2	3.35	.001
Left basolateral amygdala							
Posterior Cingulum	R	4	-40	26	8	3.63	<.001
Superior Frontal Gyrus	L	-22	40	50	1	3.50	.001
Middle Frontal Gyrus	R	34	32	32	2	3.42	.001
Middle Frontal Gyrus	L	-52	48	20	1	3.34	.001
Right basolateral amygdala							
No suprathreshold clusters							
Suppression							
Left centromedial amygdala							
Inferior Frontal Gyrus Triangularis	L	-52	28	22	107	4.22	<.001
Middle Cingulum	L	-18	2	36	5	4.13	<.001
Inferior Frontal Gyrus Opercularis	R	52	16	32	38	3.84	<.001
Inferior Frontal Gyrus Triangularis	R	52	38	16	8	3.80	<.001
Inferior Frontal Gyrus Triangularis	R	46	28	28	14	3.77	<.001
Inferior Orbitofrontal Gyrus	L	-40	30	-20	2	3.40	.001
Precentral Gyrus	R	44	0	38	2	3.40	.001
Precentral Gyrus	L	-48	10	30	3	3.36	.001
Right centromedial amygdala							
Superior Frontal Gyrus	R	18	64	32	4	3.79	<.001
Middle Cingulum	L	-18	4	34	4	3.64	<.001
Middle Frontal Gyrus	L	-50	50	20	2	3.60	<.001
Middle Frontal Gyrus	R	32	50	34	3	3.57	.001
Caudate	L	-16	2	30	2	3.45	.001
Middle Frontal Gyrus	R	46	32	30	12	3.45	.001
Middle Frontal Gyrus	L	-38	52	24	3	3.44	.001
Middle Frontal Gyrus	R	28	64	24	1	3.41	.001
Supplementary Motor Area	R	4	-2	64	2	3.41	.001
Middle Frontal Gyrus	R	48	36	36	1	3.36	.001
Left basolateral amygdala							

Inferior Orbitofrontal Gyrus	R	58	40	-4	11	4.69	<.001
Middle Frontal Gyrus	R	46	28	32	6	3.53	.001
Middle Frontal Gyrus	R	34	26	40	2	3.43	.001
Superior Frontal Gyrus	L	-12	70	14	1	3.42	.001
Middle Frontal Gyrus	R	50	58	6	1	3.40	.001
Right basolateral amygdala							
Superior Orbitofrontal Gyrus	R	16	46	-30	2	3.76	<.001
Superior Orbitofrontal Gyrus	R	20	44	-28	3	3.53	.001
Inferior Frontal Gyrus Triangularis	R	58	42	0	1	3.36	.001
Middle Cingulum	R	20	-32	42	1	3.33	.001

Note. Significance threshold for seed-to-voxel analyses set at $p < .001$ uncorrected. Coordinates are given in MNI space. Amy, amygdala; R, right; L, left; H, Hemisphere.

Supplementary Table S13. Significant clusters associated with experiential reappraisal success with respective amygdala seeds for experiment 3, restricted to PFC mask (aim 2)

Region	H	x	y	*z*	*k*	*T/F*	*p*-uncorr
Left amygdala (BLA + CMA)							
no suprathreshold clusters							
Right amygdala (BLA + CMA)							
Inferior Frontal Gyrus Triangularis	R	62	24	-2	9	3.76	<.001
Inferior Orbitofrontal Gyrus	R	62	22	-6	1	3.51	.001
Insula	R	28	28	-2	1	3.37	.001
Inferior Orbitofrontal Gyrus	L	-48	26	-4	1	3.36	.001
Amygdala (Any nucleus)							
Inferior Frontal Gyrus Triangularis	R	62	22	0	27	7.43	<.001
Insula	R	40	20	0	31	6.27	<.001
Rolandic Operculum	L	-28	-32	18	2	5.66	<.001
Middle Orbitofrontal Gyrus	L	-24	44	-22	4	5.60	<.001
Insula	R	32	26	2	5	5.60	<.001
Middle Orbitofrontal Gyrus	R	34	48	-16	7	5.38	<.001
Precuneus	L	-2	-64	28	2	5.15	.001
Insula	R	28	26	-2	1	5.11	.001
Precuneus	L	-12	-48	26	4	5.10	.001
Olfactory Gyrus	R	10	8	-18	2	4.97	.001

Note. Significance threshold for seed-to-voxel analyses set at $p < .001$ uncorrected. Coordinates are given in MNI space. Amy, amygdala; R, right; L, left; H, Hemisphere.

Supplementary Table S14. Significant clusters associated with neuronal reappraisal success for amygdala as seeds for experiment 3, restricted to PFC mask (aim 3)

Region	H	x	y	*z*	*k*	*F*	*p*-uncorr
Amygdala (Any nucleus)							
Middle Cingulum	L	-14	-28	34	7	6.74	<.001
Medial Orbitofrontal Gyrus	L	-6	32	-14	12	5.71	<.001

Medial Orbitofrontal Gyrus	R	2	42	-14	5	5.61	<.001
Medial Orbitofrontal Gyrus	L	-2	44	-14	4	5.54	<.001
Middle Cingulum	R	8	-28	44	2	5.32	.001
Medial Superior Frontal Gyrus	L	-6	62	28	5	5.20	.001
Precentral Gyrus	R	44	0	48	1	5.18	.001

Note. Significance threshold for seed-to-voxel analyses set at $p < .001$ uncorrected. Coordinates are given in MNI space. Amy, amygdala; R, right; L, left; H, Hemisphere.

Whole sample without covariate

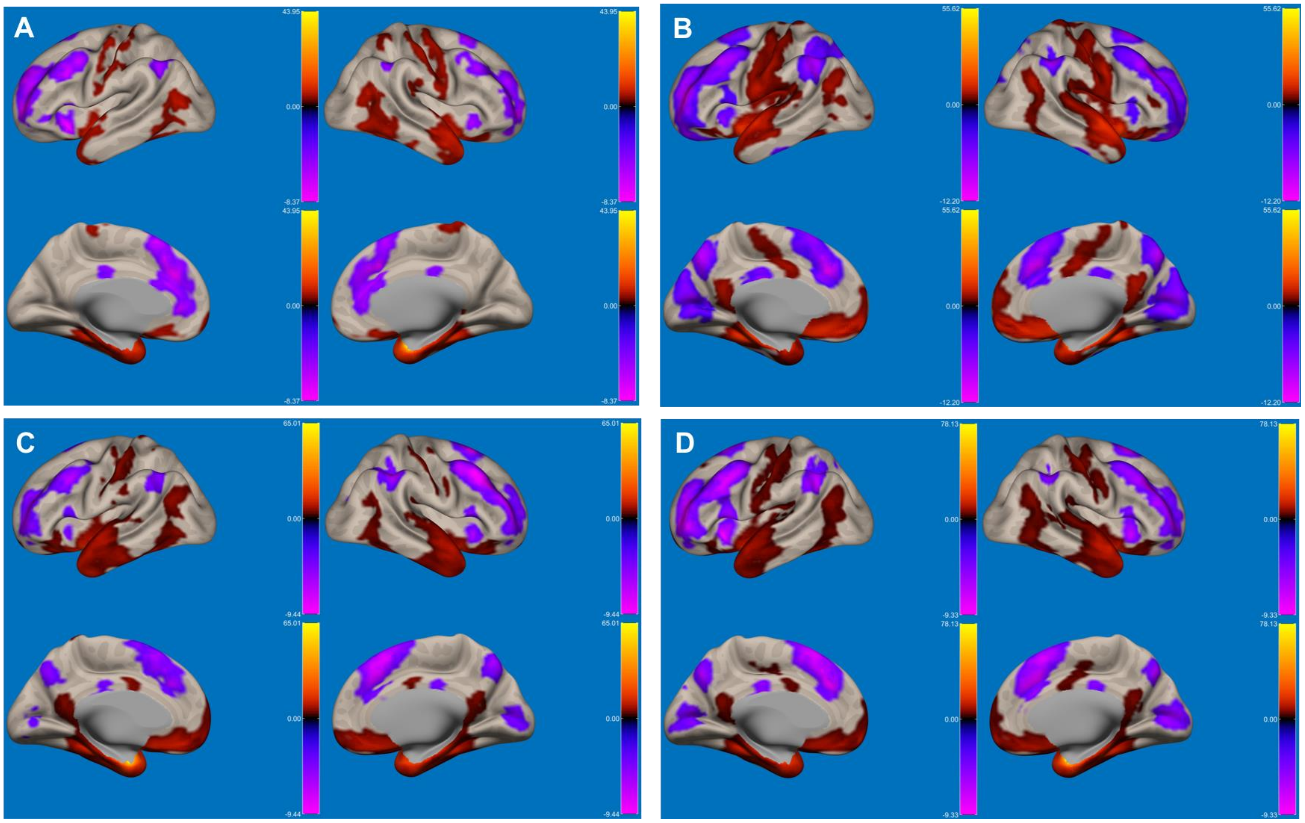

Supplementary Figure S5. Basic whole-brain seed-to-voxel connectivity maps during resting state for the whole sample, not restricted to PFC mask, without covariate and regressors. Blobs depict regions positively (red) or negatively (blue) coupled with left centromedial amygdala (A), right centromedial amygdala (B), left basolateral amygdala (C), and right basolateral amygdala (D). Results are presented on a voxel-level of $p < 0.001$ uncorrected, FWE cluster-level corrected for multiple comparisons ($p < 0.05$). Connectivity maps are presented on a rendered brain surface from CONN.

Supplementary Table S15. Significant clusters associated with the four amygdala nuclei as seeds for the whole sample, not restricted to PFC mask (whole brain analyses), without covariate and regressors

Region	H	x	y	z	k	T	p-FWE
Left centromedial amygdala							
Amygdala/Caudate Nucleus	L/R	-18	-4	-18	26275	98.51	<.001
Precentral Gyrus/Postcentral Gyrus	R	56	-10	42	871	8.09	<.001
Rolandic Operculum	R	38	-16	20	348	7.93	<.001
Middle Cingulum	R/L	0	0	36	260	7.87	<.001
Angular Gyrus/Middle Temporal Gyrus	L	-38	-62	22	451	7.53	<.001

Middle Occipital Gyrus/Middle Temporal Gyrus	R	56	-66	24	268	7.30	<.001
Inferior Frontal Gyrus Triangularis	R	54	36	8	51	6.34	<.001
Superior Frontal Gyrus	L	-14	38	48	24	6.13	<.001
Thalamus	R	0	-10	6	26	6.09	<.001
Superior Occipital Gyrus	L	-8	-108	8	36	6.07	<.001
Supplementary Motor Area	R	12	-8	46	15	6.01	<.001
Superior Temporal Gyrus	R	62	-22	4	62	5.97	<.001
Middle Temporal Gyrus	R	52	-68	-2	86	5.89	<.001
Superior Temporal Gyrus	L	-64	-26	8	18	5.80	<.001
Superior Occipital Gyrus	R	18	-106	4	7	5.70	<.001
Middle Temporal Gyrus	L	-56	-36	8	9	5.57	<.001
Inferior Temporal Gyrus	R	40	-58	-8	5	5.48	<.001
Supramarginal Gyrus	L	-58	-26	26	11	5.48	<.001
Precentral Gyrus	R	36	-26	68	13	5.41	<.001
Middle Occipital Gyrus	R	30	-100	6	2	5.23	<.001
Middle Occipital Gyrus	R	34	-100	0	1	5.13	<.001
Right centromedial amygdala							
Hippocampus/Amygdala/Superior Temporal Pole	R	18	-4	-16	27197	105.22	<.001
Middle Temporal Gyrus	R	50	-62	22	368	7.95	<.001
Inferior Orbitofrontal Gyrus	L	-28	32	-14	94	7.94	<.001
Middle Cingulum	R	0	0	36	211	6.40	<.001
Middle Temporal Gyrus/Angular Gyrus	L	-40	-64	20	300	6.33	<.001
Inferior Frontal Gyrus Triangularis	R	52	36	10	39	6.20	<.001
Cerebellum Crus1	L	-38	-88	-30	8	5.84	<.001
Putamen	R	26	-8	12	20	5.42	<.001
Postcentral Gyrus	R	14	-38	72	4	5.30	<.001
Postcentral Gyrus	R	38	-32	68	3	5.18	<.001
Precentral Gyrus	R	34	-22	66	4	5.18	<.001
Left basolateral amygdala							
Amygdala/Hippocampus	L	-22	-2	-26	23105	72.46	<.001
Postcentral Gyrus/Inferior Parietal Gyrus	L	-54	-30	58	222	7.00	<.001
Middle Cingulum	R	2	0	34	29	6.33	<.001
Middle Temporal Gyrus	L	-40	-66	20	63	6.05	<.001
Inferior Frontal Gyrus Triangularis	R	54	38	10	20	6.03	<.001
Cerebellum 3	R	14	-40	-24	19	5.80	<.001
Inferior Temporal Gyrus	R	52	-64	-6	31	5.51	<.001
Middle Temporal Gyrus	R	52	-62	16	16	5.47	<.001
Precentral Gyrus	R	52	-12	56	6	5.39	<.001
Postcentral Gyrus	R	58	-18	52	2	5.23	<.001
Inferior Temporal Gyrus	R	62	-30	-30	1	5.22	<.001
Inferior Temporal Gyrus	R	60	-32	-32	1	5.19	<.001
Middle Temporal Gyrus	L	-60	-30	6	1	5.15	<.001
Superior Frontal Gyrus	L	-10	68	22	1	5.13	<.001
Right basolateral amygdala							
Amygdala/Hippocampus/Middle Temporal Pole	R	26	0	-24	23954	96.67	<.001
Postcentral Gyrus	R	56	-18	52	847	7.24	<.001
Middle Temporal Gyrus	L	-40	-66	20	128	7.05	<.001

Postcentral Gyrus	L	-56	-18	48	257	7.01	<.001
Postcentral Gyrus/Precentral Gyrus	L	-40	-32	66	93	5.99	<.001
Inferior Occipital Gyrus	L	-52	-68	-4	51	5.70	<.001
Postcentral Gyrus	L	-40	-22	42	33	5.69	<.001
Superior Temporal Gyrus	L	-42	-20	-2	12	5.64	<.001
Cerebellum Crus1	L	-38	-88	-30	3	5.61	<.001
Postcentral Gyrus	L	-60	-8	16	23	5.44	<.001
Middle Cingulum	R	0	-2	36	8	5.33	<.001
Middle Frontal Gyrus	L	-26	38	56	3	5.32	<.001
Rolandic Operculum	L	-34	-34	18	3	5.31	<.001
Postcentral Gyrus	L	-50	-14	34	4	5.28	<.001
Vermis 10	L	-6	-50	-28	2	5.25	<.001
Inferior Temporal Gyrus	L	-62	-30	-26	1	5.22	<.001

Note. Significance threshold for seed-to-voxel analyses set at $p < .05$ FWE corrected. Coordinates are given in MNI space. Amy, amygdala; R, right; L, left; H, Hemisphere.

Supplementary Table S16. Significant clusters associated with the four amygdala nuclei as seeds for the whole sample, restricted to PFC mask, without covariate, for reappraisal and suppression (aim 1)

Region	H	x	y	*z*	*k*	*T*	*p*-uncorr
Reappraisal							
Left centromedial amygdala							
Middle Cingulum	L	-16	-34	28	2	3.30	.001
Right centromedial amygdala							
Insula	L	-34	10	-14	28	3.76	<.001
Insula	R	34	-16	22	5	3.38	.001
Middle Cingulum	L	-18	-38	32	2	3.28	.001
Middle Cingulum	L	-16	0	36	1	3.23	.001
Left basolateral amygdala							
Superior Orbitofrontal Gyrus	L	-20	34	-28	2	3.29	.001
Right basolateral amygdala							
Middle Cingulum	R	4	-38	44	43	3.88	<.001
Inferior Frontal Gyrus Triangularis	L	-54	38	20	18	3.86	<.001
Middle Frontal Gyrus	L	-38	44	28	1	3.28	.001
Suppression							
Left centromedial amygdala							
Inferior Frontal Gyrus Opercularis	R	60	16	38	15	3.90	<.001
Inferior Orbitofrontal Gyrus	L	-30	20	-24	2	3.65	<.001
Superior Medial Frontal Gyrus	R	14	60	30	12	3.56	<.001
Inferior Orbitofrontal Gyrus	L	-14	12	-26	1	3.45	<.001
Middle Cingulum	L	-8	-46	34	10	3.28	.001
Right centromedial amygdala							
Middle Frontal Gyrus	L	-28	20	44	31	4.23	<.001
Superior Frontal Gyrus	R	16	28	40	15	3.87	<.001
Angular Gyrus	L	-38	-46	20	2	3.73	<.001
Superior Medial Frontal Gyrus	L	-12	38	22	4	3.44	<.001

Middle Cingulum	L	-8	-4	32	2	3.33	.001
Inferior Orbitofrontal Gyrus	L	-30	20	-24	1	3.30	.001
Middle Cingulum	L	-20	-26	40	1	3.28	.001
Left basolateral amygdala							
Inferior Frontal Gyrus Opercularis	R	56	16	36	27	3.66	<.001
Superior Temporal Pole	L	-40	20	-18	7	3.58	<.001
Middle Frontal Gyrus	R	54	52	8	1	3.29	.001
Gyrus Rectus	R	12	32	-20	1	3.22	.001
Right basolateral amygdala							
Superior Frontal Gyrus	R	18	24	40	22	3.98	<.001
Superior Frontal Gyrus	L	-16	42	32	8	3.51	<.001
Middle Cingulum	L	-18	-22	38	4	3.46	<.001
Gyrus Rectus	R	4	30	-16	1	3.20	.001

Note. Significance threshold for seed-to-voxel analyses set at $p < .001$ uncorrected. Coordinates are given in MNI space. Amy, amygdala; R, right; L, left; H, Hemisphere.

Supplementary Table S17. Significant clusters associated with experiential reappraisal success with respective amygdala seeds for the whole sample, restricted to PFC mask, without covariate (aim 2)

Region	H	x	y	*z*	*k*	*T/F*	*p*-uncorr
Left amygdala (BLA + CMA)							
Middle Cingulum	L	-16	10	34	45	4.47	<.001
Middle Orbitofrontal Gyrus	L	-32	48	-2	18	3.98	<.001
Inferior Frontal Gyrus Opercularis	L	-40	10	10	6	3.45	<.001
Middle Frontal Gyrus	L	-32	24	30	2	3.26	.001
Right amygdala (BLA + CMA)							
Middle Cingulum	L	-10	12	32	46	4.28	<.001
Inferior Frontal Gyrus Opercularis	R	42	8	30	58	3.98	<.001
Inferior Frontal Gyrus Triangularis	L	-58	42	8	1	3.56	<.001
Inferior Orbitofrontal Gyrus	R	48	42	-10	32	3.47	<.001
Inferior Frontal Gyrus Triangularis	R	42	32	6	7	3.36	.001
Middle Cingulum	R	8	-2	34	1	3.28	.001
Middle Cingulum	L	-14	24	30	1	3.28	.001
Middle Frontal Gyrus	R	38	4	38	4	3.28	.001
Amygdala (Any nucleus)							
Middle Frontal Gyrus	R	44	4	62	7	6.14	<.001
Paracentral Lobule	L	-14	-18	64	7	6.02	<.001
Superior Frontal Gyrus	R	22	44	54	1	5.18	<.001
Middle Frontal Gyrus	L	-26	38	52	5	5.12	<.001
Middle Cingulum	L	-16	8	34	4	5.08	.001
Inferior Frontal Gyrus Triangularis	L	-58	42	8	1	5.05	.001
Middle Frontal Gyrus	L	-34	50	2	1	4.97	.001
Middle Cingulum	L	-14	20	30	1	4.96	.001
Middle Frontal Gyrus	L	-38	46	2	1	4.91	.001
Middle Frontal Gyrus	L	-32	22	30	2	4.89	.001

Insula	L	-34	20	4	1	4.78	.001
Middle Frontal Gyrus	L	-30	34	52	1	4.75	.001
Supplementary Motor Area	R	14	14	58	1	4.73	.001
Superior Frontal Gyrus	R	22	-2	70	1	4.73	.001
Inferior Frontal Gyrus Opercularis	R	40	16	8	2	4.72	.001

Note. Significance threshold for seed-to-voxel analyses set at $p < .001$ uncorrected. Coordinates are given in MNI space. Amy, amygdala; R, right; L, left; H, Hemisphere.

Supplementary Table S18. Significant clusters associated with neuronal reappraisal success for amygdala as seeds for the whole sample, restricted to PFC mask, without covariate

Region	H	x	y	*z*	*k*	*F*	*p*-uncorr
Amygdala (Any nucleus)							
Superior Frontal Gyrus	R	16	48	48	9	5.25	<.001

Note. Significance threshold for seed-to-voxel analyses set at $p < .001$ uncorrected. Coordinates are given in MNI space. Amy, amygdala; R, right; L, left; H, Hemisphere.

Whole sample with PFC mask and covariate (cf. manuscript)

Supplementary Table S19. Significant clusters associated with the four amygdala nuclei as seeds for the whole sample, with experiment as covariate, restricted to PFC mask

Region	H	x	y	z	*k*	*T*	*p*-uncorr
Left centromedial amygdala							
Superior Orbitofrontal Gyrus	L	-12	20	-24	115	4.59	<.001
Superior Frontal Gyrus	L	-16	56	22	41	4.43	<.001
Supplementary Motor Area	R	10	-6	82	11	3.97	<.001
Inferior Orbitofrontal Gyrus	L	-42	28	-14	51	3.81	<.001
Middle Orbitofrontal Gyrus	R	38	38	-14	13	3.38	.001
Medial Orbitofrontal gyrus	L	-8	38	-10	2	3.33	.001
Supplementary Motor Area	R	16	-6	50	2	3.29	.001
Inferior Frontal Gyrus Opercularis	R	36	4	28	1	3.25	.001
Middle Cingulum	L	-16	10	34	1	3.24	.001
Right centromedial amygdala							
Middle Orbitofrontal Gyrus	L	-26	38	-6	20	3.89	<.001
Superior Frontal Gyrus	R	16	48	26	10	3.62	<.001
Superior Frontal Gyrus	L	-26	-8	66	8	3.61	<.001
Inferior Frontal Gyrus Triangularis	R	44	32	8	28	3.55	<.001
Insula	L	-34	22	8	22	3.49	<.001
Rolandic Operculum	L	-46	-24	14	11	3.42	<.001
Insula	R	28	32	-2	2	3.35	.001
Inferior Orbitofrontal Gyrus	R	40	22	-20	1	3.25	.001
Rolandic Operculum	L	-28	-30	16	1	3.21	.001
Left basolateral amygdala							
Superior Orbitofrontal Gyrus	L	-12	20	-24	115	4.59	<.001
Superior Frontal Gyrus	L	-16	56	22	41	4.43	<.001
Supplementary Motor Area	R	10	-6	82	11	3.97	<.001
Inferior Orbitofrontal Gyrus	L	-42	28	-14	51	3.81	<.001
Middle Orbitofrontal Gyrus	R	38	38	-14	13	3.38	.001
Medial Orbitofrontal Gyrus	L	-8	38	-10	2	3.33	.001
Supplementary Motor Area	R	16	-6	50	2	3.29	.001
Inferior Frontal Gyrus Opercularis	R	36	4	28	1	3.25	.001
Middle Cingulum	L	-16	10	34	1	3.24	.001
Right basolateral amygdala							
Supplementary Motor Area	R	16	-6	50	21	4.22	<.001
Supplementary Motor Area	R	10	-2	82	13	3.73	<.001
Superior Orbitofrontal Gyrus	R	18	28	-20	6	3.47	<.001
Olfactory Gyrus	L	-6	10	-20	6	3.46	<.001
Superior Orbitofrontal Gyrus	L	-12	26	-30	3	3.39	<.001
Heschl Gyrus	R	44	-18	4	3	3.25	.001
Superior Frontal Gyrus	R	22	-4	64	1	3.25	.001
Inferior Frontal Gyrus Triangularis	R	42	30	6	1	3.22	.001

Note. Significance threshold for seed-to-voxel analyses set at $p < .001$ uncorrected. Coordinates are given in MNI space. Amy, amygdala; R, right; L, left; H, Hemisphere.

Supplementary Table S20. Significant clusters associated with the four amygdala nuclei as seeds for the whole sample, with experiment as covariate, restricted to PFC mask for reappraisal and suppression (aim 1)

Region	H	x	y	z	*k*	*T*	*p*-uncorr
Reappraisal							
Left centromedial amygdala							
Middle Cingulum	L	-16	-34	30	2	3.30	.001
Right centromedial amygdala							
Insula	L	-34	12	-14	49	4.16	<.001
Insula	R	34	-16	22	1	3.28	.001
Middle Cingulum	L	-16	0	36	3	3.26	.001
Insula	R	34	-20	22	1	3.24	.001
Left basolateral amygdala							
No suprathreshold clusters							
Right basolateral amygdala							
Middle Cingulum	R	4	-38	44	41	3.79	<.001
Inferior Frontal Gyrus Triangularis	L	-54	38	20	10	3.64	<.001
Middle Frontal Gyrus	L	-38	44	28	7	3.38	.001
Middle Frontal Gyrus	R	32	38	22	1	3.23	.001
Suppression							
Left centromedial amygdala							
Superior Medial Frontal Gyrus	R	14	58	30	17	3.76	<.001
Inferior Frontal Gyrus Opercularis	R	60	16	38	10	3.66	<.001
Middle Cingulum	L	-8	-44	32	29	3.50	<.001
Inferior Frontal Gyrus Orbicularis	L	-30	20	-24	1	3.43	<.001
Superior Temporal Gyrus	L	-40	22	-18	2	3.33	.001
Inferior Frontal Gyrus Orbicularis	L	-14	12	-26	1	3.33	.001
Right centromedial amygdala							
Middle Frontal Gyrus	L	-30	20	44	34	4.30	<.001
Superior Frontal Gyrus	R	18	26	40	14	3.75	<.001
Middle Cingulum	L	-8	-4	32	4	3.44	<.001
Angular Gyrus	L	-38	-46	20	1	3.44	<.001
Superior Medial Frontal Gyrus	L	-12	38	22	4	3.40	<.001
Anterior Cingulum	R	8	48	20	5	3.30	.001
Left basolateral amygdala							
Superior Temporal Gyrus	L	-40	20	-16	11	3.67	<.001
Inferor Frontal Gyrus Opercularis	R	56	16	36	15	3.50	<.001
Middle Frontal Gyrus	R	54	52	8	1	3.26	.001
Gyrus Rectus	R	12	32	-20	1	3.19	.001
Right basolateral amygdala							
Superior Frontal Gyrus	R	18	24	40	23	4.29	<.001
Middle Cingulum	L	-18	-26	38	6	3.56	<.001
Superior Frontal Gyrus	L	-16	42	32	8	3.48	<.001

Middle Cingulum	L	-8	-2	32	3	3.34	.001

Note. Significance threshold for seed-to-voxel analyses set at *p* < .001 uncorrected. Coordinates are given in MNI space. Amy, amygdala; R, right; L, left; H, Hemisphere.

Supplementary Table S21. Significant clusters associated with experiential reappraisal success with respective amygdala seeds for the whole sample, with experiment as covariate, restricted to PFC mask (aim 2)

Region	H	x	y	z	*k*	*T/F*	*p*-uncorr
Left amygdala (BLA + CMA)							
Middle Cingulum	L	-14	8	32	17	3.76	<.001
Inferior Frontal Gyrus Opercularis	L	-38	10	12	19	3.67	<.001
Middle Frontal Gyrus	L	-32	48	0	3	3.26	.001
Middle Frontal Gyrus	L	-32	26	30	1	3.24	.001
Rolandic Operculum	R	42	-4	16	1	3.20	.001
Right amygdala (BLA + CMA)							
Middle Cingulum	L	-12	14	32	21	3.88	<.001
Precentral Gyrus	R	46	6	28	39	3.63	<.001
Inferior Frontal Gyrus Triangularis	L	-58	42	8	1	3.23	.001
Amygdala (Any nucleus)							
Paracentral Lobule	L	-14	-18	64	-14	5.32	<.001
Paracentral Lobule	L	-8	-30	64	-8	5.11	.001

Note. Significance threshold for seed-to-voxel analyses set at *p* < .001 uncorrected. Coordinates are given in MNI space. Amy, amygdala; R, right; L, left; H, Hemisphere.

Supplementary Table S22. Significant clusters associated with neuronal reappraisal success for amygdala nuclei as seeds for the whole sample, with experiment as covariate, restricted to PFC mask (aim 3)

Region	H	x	y	z	*k*	*F*	*p*-uncorr
Amygdala (Any nucleus)							
Superior Frontal Gyrus	R	16	48	48	12	5.52	<.001
Medial Orbitofrontal Gyrus	R	2	58	-14	2	4.78	.001
Middle Frontal Gyrus	R	46	8	56	1	4.73	.001

Note. Significance threshold for seed-to-voxel analyses set at *p* < .001 uncorrected. Coordinates are given in MNI space. Amy, amygdala; R, right; L, left; H, Hemisphere.

Differences in methods

Supplementary Table S23. Differences in methods between (Pico-Perez et al., 2018) and the replication (for details see also https://osf.io/8wsgu)

	**Pico-Perez et al. (2018)**	**Replication**
Participants		
Sample Size	Participated = not reported<br>Analyzed: N = 48	Participated: N = 136<br>Analyzed: N = 107
Demographics	23 female<br>age: 39.6 (SD = 9.64), range 19-56	64 female<br>age: 24.4 (SD = 4.2), range: 18-48
Population	Not reported	University community in Germany, healthy young adults
Exclusion/ Inclusion criteria	Exclusion<br>• Presence or past history (in the previous 6 months) of psychoactive substance abuse or dependence<br>• intellectual disability<br>• presence or past history of any severe medical condition<br>• any MRI contraindication	Exclusion<br>• MRI contraindications<br>• current or prior medical, neurological or psychiatric illness or treatment<br>• left-handedness
Procedure		
Procedure	ERQ, structural and RS-fMRI performed on the same day.<br>No detailed procedure reported	2 sessions one week apart<br>Session 1: ERT-fMRI, structural MRI<br>Session 2: RS-fMRI, ERQ
Psychometric measurements	Spanish version of ERQ (Cabello, Salguero, Fernández-Berrocal, & Gross, 2013)<br>Reliability:<br>$\alpha_{reappraisal} = 0.72$<br>$\alpha_{suppression} = 0.71$	German version of ERQ (Abler & Kessler, 2009)<br>Reliability:<br>$\alpha_{reappraisal} = 0.74$<br>$\alpha_{suppression} = 0.76$
Experimental design	None	ER-Task
fMRI acquisition (RS-fMRI)		
System	GE 1.5 Tesla	Siemens Magnetom Trio 3.0 Tesla
fMRI sequence	GRASS	EPI

Echo time (TE)	50 ms	25 ms
Repetition time (TR)	2000 ms	2410 ms
Flip angle	90°	80°
Field of view	240 mm	192 mm
Slice Thickness	4.00 mm	2.00 mm
Number of slices	22	42
Gap	1.00 mm	1.00 mm
Matrix size	64 × 64 mm<br>3.75 × 3.75 × 4	64 × 64 mm<br>3 × 3 × 2 mm^3
Preprocessing		
Software	• Microsoft Windows platform<br>• MATLAB version 7 (R2012a)<br>• Statistical parametric mapping software (SPM8 sv6313)	• Windows 10 Enterprise 2016 LTSB, 64-bit<br>• MATLAB (R2019b)<br>• Statistical parametric mapping software (SPM12, v7487)<br>• CONN toolbox (version 18b)
Motion correction	aligning (within participant) each time series to the mean image volume	aligning each time series to the mean image volume using a least-squares minimization and a 6-parameter (rigid body) spatial transformation and unwarping
Coregistration	realigned functional sequences were then coregistered to each participant's respective anatomical scan that had been previously coregistered to the SPM-T1 template	a separate normalization (non-linear transformation to MNI space) of the structural and functional data according to the default preprocessing pipeline (direct normalization to MNI-space), thus, structural and functional data end up in the same space (in MNI), without having been explicitly co-registered to each other
Normalization	DARTEL<br>2 × 2 × 2	DARTEL<br>2 × 2 × 2
Outlier detection	Not reported	ART-based scrubbing
Smoothing	8 mm Gaussian kernel	8 mm Gaussian kernel
Denoising	Not reported	Yes, anatomical CompCor method (Behzadi, Restom, Liau, & Liu, 2007)
Seed extraction		

Software	MarsBaR region-of-interest toolbox	SPM Anatomy toolbox v.2.2c (Eickhoff et al., 2005)
ROI	3.5 mm radial spheres centered at: left basolateral amygdala (x = − 26, y = − 5, z = − 23), right basolateral amygdala (x = 29, y = − 3, z = − 23), left centromedial amygdala1 (x = − 19, y = − 5, z = − 15) and right centromedial amygdala (x = 23, y = − 5, z = − 13) (Fig. 1).<br>spatially separated between each other by at least 8 mm (1 FWHM)	based on Baur, Hanggi, Langer, and Jancke (2013): separate maximum probability maps were created for left basolateral amygdala and right basolateral amygdala, left centromedial amygdala and right centromedial amygdala (each including superficial divisions)
First level analyses		
Software		• MATLAB (R2019b)<br>• Statistical parametric mapping software (SPM12, v7487)<br>• CONN toolbox (version 18b)
GLM	general lineal model (GLM) including the two noise-cleaned amygdala-seed time series per hemisphere as predictors, and, as nuisance covariates, the three translation and three rotation estimates from the movement correction step plus three covariates corresponding to the white matter, cerebrospinal fluid (CSF) and whole brain signal estimates	general lineal model (GLM) including the four noise-corrected amygdala-seed time series as predictors as well as the 6 movement parameters and ART-detected outliers as first-level nuisance covariates of no interest
High pass filter	0.008 Hz at 128 s	0.008–0.09 Hz
Second level analyses		
Software	Statistical parametric mapping software (SPM8 sv6313)?<br>SPM-TFCE toolbox<br>v117 (http://dbm.neuro.uni-jena.de/tfce/)	• MATLAB (R2019b)<br>• Statistical parametric mapping software (SPM12, v7487)<br>• CONN toolbox (version 18b)
GLM		ERQ subscales reappraisal and suppression served as predictors of interest, experiment (1,2,3) as covariate
ROI	17,391-voxel mask created with the Wake Forest University (WFU) Pick-atlas toolbox (Maldjian, Laurienti, Kraft, & Burdette, 2003)<br>comprising different regions of the frontal lobe (i.e., inferior frontal, middle frontal, superior frontal, medial frontal and orbital gyri), the cingulate gyri and the insulae	56,833-voxel mask ($2 \times 2 \times 2$ mm^3) created with the Wake Forest University (WFU) Pick-atlas toolbox (Maldjian et al., 2003) comprising different regions of the frontal lobe (i.e., inferior frontal, middle frontal, superior frontal, medial frontal and orbital gyri), the cingulate gyri and the insulae.
Statistical thresholds	$p < 0.05$, Family-Wise Error (FWE) corrected (ROI), voxel-wise non-parametric permutation testing (Nichols and Holmes 2001) with 5000 permutations was performed using	For all analyses, the significance threshold was set to $p < .05$, family-wise error corrected (FWE) for multiple comparisons.

	the Threshold-Free Cluster Enhancement (TFCE) technique (Smith and Nichols 2009)	

Supplementary Table S24. Differences in methods between (Uchida et al., 2015) and the replication (for details see also https://osf.io/8wsgu)

	**Uchida et al. (2015)**	**Replication**
Participants		
Sample Size	Participated: N = 72<br>Analyzed: N = 62	Participated: N = 136<br>Analyzed: N = 107
Demographics	32 female<br>Age: 22.3 (SD = 1.6)	64 female<br>age: 24.4 (SD = 4.2), range: 18-48
Population	community recruited through advertising in the local media	University community in Germany, healthy young adults
Exclusion/ Inclusion criteria	Exclusion<br>• major sensorimotor handicaps (paralysis, deafness, blindness)<br>• history of psychosis<br>• autism<br>• currently taking psychiatric medication,<br>• inadequate command of the English language<br>• IQ below 80,<br>• any conditions incompatible with Magnetic Resonance Imaging (MRI) scanning<br>• positive pregnancy test<br>• history of traumatic head injuries<br>Inclusion<br>Equal numbers of participants were selected to score below 53, 54–72 or above 73 points on the DERS (Gratz & Roemer, 2004)	Exclusion<br>• MRI contraindications<br>• current or prior medical, neurological or psychiatric illness or treatment<br>• left-handedness
Procedure		
Procedure	ERT-fMRI, RS-fMRI, PANAS-X, STAI-T on the same day. No detailed procedure reported	2 sessions one week apart<br>Session 1: 1. ERT-fMRI, 2. structural MRI, re-exposure fMRI

		Session 2: 1. RS-fMRI, 2. Questionnaires<br>Details at https://osf.io/8wsgu
Psychometric measurements	Not relevant	Not relevant
Experimental design	ER-Task<br>3 conditions: attend-neutral, attend-negative, reappraise-negative<br>Attend: “attend to the picture, by naturally experiencing the emotional state elicited by the picture”<br>Reappraise: “reappraise, whereby participants reinterpreted the picture in an effort to reduce their negative feelings about it.”	ER-Task<br><br>Experiment 1:<br>Regulation (3) × stimulus valence (2) (not fully balanced), within subject<br>Permit-negative, detach-negative, view-negative, view-neutral<br><br>Experiment 2:<br>Regulation (2) × stimulus valence (2) (fully balanced), within subject<br>Permit-neutral, permit-negative, detach-neutral, detach-negative<br><br>Experiment 3:<br>Regulation (3) × stimulus valence (2) (fully balanced), within subject<br>Permit-neutral, permit-negative, detach-neutral, detach-negative, intensify-neutral, intensify-negative<br><br>see https://osf.io/8wsgu for instructions in the conditions
Trial	Instructions (2sec), anticipatory interval (4sec), image (8sec), inter-stimulus interval (4 or 7 sec), response (negative emotional reaction, 2.9sec), inter-stimulus interval (4 or 7sec)	Experiment 1<br>stimulation period (picture, 8sec, initial 2sec instruction with semi-transparent overlay), a rating period (3sec), relaxation period (12sec), a rating period (3sec), variable interval with a mean duration of 4s<br><br>Experiment 2<br>stimulation period (picture, 10sec, initial 2s instruction with a semi-transparent overlay), relaxation period (16 – 24 secs) retrospective arousal ratings for each condition after each run<br><br>Experiment 3

		stimulation period (picture, 10sec, initial 2s instruction with a semi-transparent overlay), relaxation period (12 – 20 secs) retrospective arousal ratings for each condition after each run
Stimuli	60 stimuli from the IAPS (Lang, Bradley, & Cuthbert, 2008)<br>20 neutral: valence mean = 5.1, arousal mean = 3.3<br>40 negative: set A: valence mean = 2.2, arousal mean = 5.7);<br>set B: valence mean = 2.2, arousal mean = 5.7	negative (categories: animal, body, disaster, disgust, injury, suffering, violence, and weapons) and neutral (categories: objects, persons, and scenes) pictures from the International Affective Picture System (IAPS) (Lang et al., 2008) and the Emotional Picture Set (EmoPicS) (Wessa et al., 2010).<br>negative:<br>80 pictures in experiment 1, valence ratings (V) = 2.67-2.81, arousal ratings (A) = 5.54-5.74<br>32 pictures in experiment 2, V = 2.65-2.71 and A = 5.69-5.85<br>48 pictures in experiment 3, V = 2.65-2.71 and A = 5.55-5.85<br>neutral:<br>40 pictures in experiment 1, V = 4.98-5.16 and A = 2.86-3.04<br>32 pictures in experiment 2, V = 5.13-5.17 and A = 2.94-2.96<br>48 pictures in experiment 3, V = 5.13-5.19 and A = 2.85-2.96
fMRI acquisition (RS-fMRI)		
System	Siemens Tim Trio 3.0 Tesla	Siemens Magnetom Trio 3.0 Tesla
fMRI sequence	Not reported	EPI
Echo time (TE)	30 ms	25 ms
Repetition time (TR)	6000 ms	2410 ms
Flip angle	Not reported	80°
Field of view	Not reported	192 mm
Slice Thickness	2.00 mm	2.00 mm
Number of slices	67	42
Gap	Not reported	1.00 mm
Matrix size	Not reported<br>2 × 2 × 2 mm	64 × 64 mm<br>3 × 3 × 2 mm
fMRI acquisition (ERT-fMRI)		
System	Siemens Tim Trio 3.0 Tesla	Siemens Magnetom Trio 3.0 Tesla

fMRI sequence	Not reported	EPI
Echo time (TE)	30 ms	25 ms
Repetition time (TR)	2000 ms	2410 ms
Flip angle	Not reported	80°
Field of view	Not reported	192 mm
Slice Thickness	4 mm	2.00 mm
Number of slices	Not reported	42
Gap	Not reported	1.00 mm
Matrix size	Not reported	64 × 64 mm<br>3 × 3 × 2 mm
	Preprocessing	
Software	All fMRI data:<br>• MATLAB<br>• Statistical parametric mapping software (SPM 8)	ERT-fMRI:<br>• Microsoft Windows platform<br>• MATLAB version 7.4<br>• Statistical parametric mapping software (SPM 8, SPM12, v7487)<br><br>RS-fMRI:<br>• Windows 10 Enterprise 2016 LTSB, 64-bit<br>• MATLAB (R2019b)<br>• Statistical parametric mapping software (SPM12, v7487)<br>• CONN toolbox (version 18b)
Motion correction	Yes, no details reported<br><br>RS-fMRI:<br>To address spurious correlations in resting-state networks caused by head motion, we used quality assurance software Artifact Detection Tools (http://www.nitrc.org/projects/artifact_detect)	ERT-fMRI<br>aligning each time series to the mean image volume using a least-squares minimization and a 6-parameter (rigid body) spatial transformation. Fieldmap-based unwarping<br><br>RS-fMRI:<br>aligning each time series to the mean image volume using a least-squares minimization and a 6-parameter (rigid body) spatial transformation and unwarping
Coregistration	All fMRI data: not reported	ERT-fMRI

		individual realigned mean EPI to the individual anatomical scan<br><br>RS-fMRI<br>a separate normalization (non-linear transformation to MNI space) of the structural and functional data according to the default preprocessing pipeline (direct normalization to MNI-space), thus, structural and functional data end up in the same space (in MNI), without having been explicitly co-registered to each other
Normalization	All fMRI data: Yes, no details reported	(deviating from the preregistration)<br>ERT-fMRI<br>spatial normalization of the anatomical data to the MNI template, application of the estimated transformation parameters to the coregistered functional images using a resampling resolution of 2 × 2 × 2 mm^3<br><br>RS-fMRI:<br>normalization to the MNI reference brain, 2 × 2 × 2 mm^3
Outlier detection	RS-fMRI:<br>"An image was defined as an outlier image if the head displacement in x, y or z direction was >0.5mm from the previous frame, or if the global mean intensity in the image was greater than three standard deviations from the mean image intensity for the entire resting scan."	RS-fMRI:<br>ART-based scrubbing
Smoothing	All fMRI data: Yes, no details reported	ERT-fMRI and RS-fMRI<br>8 mm Gaussian kernel
Denoising	RS-fMRI:<br>Yes, anatomical CompCor method (Behzadi et al., 2007)	RS-fMRI:<br>Yes, anatomical CompCor method (Behzadi et al., 2007)
Seed extraction (RS-fMRI)		
Software	WFU_pickatlas (Maldjian et al., 2003)	SPM Anatomy toolbox v.2.2c (Eickhoff et al., 2005)
ROI	• left and right anatomical amygdalae<br>• "left and right DLPFC seed regions based on a 10 mm sphere	based on (Baur et al., 2013): separate maximum probability maps were created for left basolateral amygdala and right basolateral amygdala, left centromedial amygdala and right centromedial amygdala (each including superficial divisions)

	• around the peak voxel of the fMRI group Reappraisal>Look Negative contrast located within BA areas 9 and 46."<br>• DMN seeds: 10mm spheres around the peak coordinates of the MPFC, PCC and right/left parietal (RLP/LLP)	
First level analyses		
Software	ERT-fMRI<br>• MATLAB<br>• Statistical parametric mapping software (SPM8)<br><br>RS-fMRI:<br>• MATLAB<br>• Statistical parametric mapping software (SPM8)<br>• CONN toolbox	ERT-fMRI<br>• Microsoft Windows platform<br>• MATLAB version 7.4<br>• Statistical parametric mapping software (SPM 8, SPM12, v7487)<br><br>RS-fMRI:<br>• MATLAB (R2019b)<br>• Statistical parametric mapping software (SPM12, v7487)<br>• CONN toolbox (version 18b)
GLM	ERT-fMRI<br>Attend Negative, Reappraise Negative, Attend Neutral individual reappraisal scores, low-frequency components of the fMRI signal were modelled as confounding covariates<br><br>RS-fMRI:<br>We performed seed-voxel correlations by estimating maps showing temporal correlations between the BOLD signal from seed regions and every other voxel in the brain.<br><br>We calculated the mean DMN resting state functional connectivity for all participants.	ERT-fMRI<br>regressors based on experimental conditions, as well as six additional motion regressors of no interest. Instructions and picture were set together as one event. Temporal patterns were modelled as boxcar function (8 s duration (experiment 1 and 3) and 10 s duration (experiment two), respectively) to cover sustained responses. All regressors were convolved with the canonical hemodynamic response function (HRF). All runs of the imaging experiments were combined within one fixed-effects model.<br><br>RS-fMRI:<br>general lineal model (GLM) including the four noise-corrected amygdala-seed time series as predictors as well as the 6 movement parameters and ART-detected outliers as first-level nuisance covariates of no interest
ROI		maximum probability maps of the left basolateral amygdala, right basolateral amygdala, left centromedial amygdala, and right

		centromedial amygdala were created using the SPM Anatomy toolbox v.2.2c (Eickhoff et al., 2005). The probability threshold was set to 40% for each voxel to provide a sufficient areal coverage of the anatomical structure (Baur et al., 2013; Eickhoff, Heim, Zilles, & Amunts, 2006)
High pass filter	Not reported	RS-fMRI<br>0.008-0.09
Second level analyses		
• Software	• MATLAB<br>• Statistical parametric mapping software (SPM8)<br>CONN toolbox	• MATLAB (R2019b)<br>• Statistical parametric mapping software (SPM12, v7487)<br>• CONN toolbox (version 18b)
GLM		1. predictors of interest: RS functional connectivity of each of the amygdala seeds, individual indices for behavioural reappraisal success (arousal ratings permit negative – detach negative), Covariate: experiment (1, 2, 3)<br><br>2. predictors of interest: RS functional connectivity of each of the amygdala seeds, individual contrast estimates from the ERT-fMRI contrast *Negative-Permit Picture > Negative Detach Picture* in the amygdala ROIs.<br>Covariate: experiment (1, 2, 3)
ROI	DMN seeds: 10mm spheres around the peak coordinates of the MPFC, PCC and right/left parietal (RLP/LLP). "Finally, we overlaid the amygdala seed based clusters, which were significantly correlated with the reappraisal success, onto the group DMN."	56,833-voxel mask ($2 \times 2 \times 2$ mm³) created with the Wake Forest University (WFU) Pick-atlas toolbox (Maldjian et al., 2003) comprising different regions of the frontal lobe (i.e., inferior frontal, middle frontal, superior frontal, medial frontal and orbital gyri), the cingulate gyri and the insulae
Statistical thresholds	All imaging analyses were corrected for multiple comparisons with an initial height threshold of $p < 0.001$ and a family wise error (FWE) cluster level corrected of $p < 0.05$.	For all analyses, the significance threshold was set to $p < .05$, family-wise error corrected (FWE) for multiple comparisons.

References

Abler, B., & Kessler, H. (2009). Emotion regulation questionnaire–A German version of the ERQ by Gross and John. *Diagnostica, 55*, 144-152.

Baur, V., Hanggi, J., Langer, N., & Jancke, L. (2013). Resting-state functional and structural connectivity within an insula-amygdala route specifically index state and trait anxiety. *Biol Psychiatry, 73*(1), 85-92. doi:10.1016/j.biopsych.2012.06.003

Behzadi, Y., Restom, K., Liau, J., & Liu, T. T. (2007). A component based noise correction method (CompCor) for BOLD and perfusion based fMRI. *NeuroImage, 37*(1), 90-101. doi:10.1016/j.neuroimage.2007.04.042

Cabello, R., Salguero, J. M., Fernández-Berrocal, P., & Gross, J. J. (2013). A Spanish Adaptation of the Emotion Regulation Questionnaire. *European Journal of Psychological Assessment, 29*(4), 234-240. doi:10.1027/1015-5759/a000150

Diers, K., Dörfel, D., Gärtner, A., Schönfeld, S., Walter, H., Strobel, A., & Brocke, B. (in preparation). Neural dynamics of cognitive emotion regulation: immediate, short- and long-term effects.

Diers, K., Weber, F., Brocke, B., Strobel, A., & Schonfeld, S. (2014). Instructions matter: a comparison of baseline conditions for cognitive emotion regulation paradigms. *Front Psychol, 5*, 347. doi:10.3389/fpsyg.2014.00347

Eickhoff, S. B., Heim, S., Zilles, K., & Amunts, K. (2006). Testing anatomically specified hypotheses in functional imaging using cytoarchitectonic maps. *NeuroImage, 32*(2), 570-582. doi:10.1016/j.neuroimage.2006.04.204

Eickhoff, S. B., Stephan, K. E., Mohlberg, H., Grefkes, C., Fink, G. R., Amunts, K., & Zilles, K. (2005). A new SPM toolbox for combining probabilistic cytoarchitectonic maps and functional imaging data. *NeuroImage, 25*(4), 1325-1335. doi:10.1016/j.neuroimage.2004.12.034

Gärtner, A., Dörfel, D., Diers, K., Witt, S. H., Strobel, A., & Brocke, B. (2019). Impact of FAAH genetic variation on fronto-amygdala function during emotional processing. *European Archives of Psychiatry and Clinical Neuroscience, 269*(2), 209-221. doi:10.1007/s00406-018-0944-9

Gratz, K. L., & Roemer, L. (2004). Multidimensional assessment of emotion regulation and dysregulation: Development, factor structure, and initial validation of the difficulties in emotion regulation scale. *Journal of Psychopathology and Behavioral Assessment, 26*(1), 41-54. doi:Doi 10.1023/B:Joba.0000007455.08539.94

Lang, P. J., Bradley, M. M., & Cuthbert, B. N. (2008). *International affective picture system (IAPS): affective ratings of pictures and instruction manual*. Gainsville, FL: University of Florida.

Maldjian, J. A., Laurienti, P. J., Kraft, R. A., & Burdette, J. H. (2003). An automated method for neuroanatomic and cytoarchitectonic atlas-based interrogation of fMRI data sets. *NeuroImage, 19*(3), 1233-1239.

Pico-Perez, M., Alonso, P., Contreras-Rodriguez, O., Martinez-Zalacain, I., Lopez-Sola, C., Jimenez-Murcia, S., . . . Soriano-Mas, C. (2018). Dispositional use of emotion regulation strategies and

resting-state cortico-limbic functional connectivity. *Brain Imaging Behav, 12*(4), 1022-1031. doi:10.1007/s11682-017-9762-3

Scheffel, C., Diers, K., Schönfeld, S., Brocke, B., Strobel, A., & Dörfel , D. (2019). Cognitive emotion regulation and personality: an analysis of individual differences in the neural and behavioral correlates of successful reappraisal. *Personality Neuroscience, 2*(e11), 1-13. doi:10.1017/pen.2019.11

Uchida, M., Biederman, J., Gabrieli, J. D. E., Micco, J., de Los Angeles, C., Brown, A., . . . Whitfield-Gabrieli, S. (2015). Emotion regulation ability varies in relation to intrinsic functional brain architecture. *Social Cognitive and Affective Neuroscience, 10*(12), 1738-1748. doi:10.1093/scan/nsv059

Wessa, M., Kanske, P., Neumeister, P., Bode, K., Heissler, J., & Schönfelder, S. (2010). EmoPics: Subjektive und psychophysiologische Evaluation neuen Bildmaterials für die klinisch-biopsychologische Forschung. *Zeitschrift für Klinische Psychologie und Psychotherapie, 39*(Suppl. 1/11), 77.
